# From Genotype to Phenotype: Nonsense Variants in *SLC13A1* Are Associated with Decreased Serum Sulfate and Increased Serum Aminotransferases

**DOI:** 10.1534/g3.116.032979

**Published:** 2016-07-13

**Authors:** Christina G. Tise, James A. Perry, Leslie E. Anforth, Mary A. Pavlovich, Joshua D. Backman, Kathleen A. Ryan, Joshua P. Lewis, Jeffrey R. O’Connell, Laura M. Yerges-Armstrong, Alan R. Shuldiner

**Affiliations:** Program for Personalized and Genomic Medicine and Division of Endocrinology, Diabetes and Nutrition, and University of Maryland School of Medicine, Baltimore, Maryland 21201

**Keywords:** *SLC13A1*, serum sulfate, loss-of-function variants, Old Order Amish, GWAS/ExWAS

## Abstract

Using genomic applications to glean insights into human biology, we systematically searched for nonsense single nucleotide variants (SNVs) that are rare in the general population but enriched in the Old Order Amish (Amish) due to founder effect. We identified two nonlinked, nonsense SNVs (R12X and W48X) in *SLC13A1* (allele frequencies 0.29% and 0.74% in the Amish; enriched 1.2-fold and 3.7-fold, compared to the outbred Caucasian population, respectively). *SLC13A1* encodes the apical sodium-sulfate cotransporter (NaS1) responsible for sulfate (re)absorption in the kidneys and intestine. *SLC13A1* R12X and W48X were independently associated with a 27.6% (*P* = 2.7 × 10^−8^) and 27.3% (*P* = 6.9 × 10^−14^) decrease in serum sulfate, respectively (*P* = 8.8 × 10^-20^ for carriers of either *SLC13A1* nonsense SNV). We further performed the first exome- and genome-wide association study (ExWAS/GWAS) of serum sulfate and identified a missense variant (L348P) in *SLC26A1*, which encodes the basolateral sulfate-anion transporter (Sat1), that was associated with decreased serum sulfate (*P* = 4.4 × 10^−12^). Consistent with sulfate’s role in xenobiotic detoxification and protection against acetaminophen-induced hepatotoxicity, *SLC13A1* nonsense SNV carriers had higher aminotransferase levels compared to noncarriers. Furthermore, *SLC26A1* L348P was associated with lower whole-body bone mineral density (BMD) and higher serum calcium, consistent with the osteochondrodysplasia exhibited by dogs and sheep with naturally occurring, homozygous, loss-of-function mutations in *Slc13a1*. This study demonstrates the power and translational potential of systematic identification and characterization of rare, loss-of-function variants and warrants additional studies to better understand the importance of sulfate in human physiology, disease, and drug toxicity.

Population isolates have been critical in elucidating the genetic basis of both classical Mendelian and complex diseases. While many loss-of-function variants associated with disease are rare in the general population, genetic drift in founder populations like the Old Order Amish (Amish) increases the probability that many individuals will share the same rare, disease-causing variant. Recent advances in genome-wide genotyping and sequencing technologies have made possible the systematic identification of these variants and their subsequent investigation.

In this study, we systematically interrogated nonsense single nucleotide variants (SNVs) from the Illumina Human Exome BeadChip for minor allele frequency (MAF) enrichment in the Amish compared to outbred, European-derived populations. Using this approach, we observed an enrichment of two nonsense SNVs (c.34G > A, p.R12X and c.144C > T, p.W48X) in *SLC13A1*, which encodes the apical membrane, sodium-sulfate cotransporter (NaS1) responsible for sulfate (re)absorption in the intestines and kidneys.

Inorganic sulfate (SO_4_^2-^) is an important micronutrient vital for numerous cellular and metabolic processes in human development and physiology (Dawson 2011, 2013; Markovich 2014). Sulfate is critical in the biotransformation of multiple compounds via sulfotransferase-mediated sulfate conjugation (sulfation) (Klaassen and Boles 1997). These compounds include hormones, neurotransmitters, proteoglycans, and xenobiotics during phase II metabolism (Dawson 2011; Markovich 2011a,b, 2014; Council 2005). Impaired sulfation capacity substantially alters the metabolism and activities of these compounds and has been implicated in several human pathologies including reduced xenobiotic clearance, skeletal dysplasia, premature pubarche, autism spectrum disorder (ASD), and neurological disease (Alberti *et al.* 1999; Bowling *et al.* 2013; Heafield *et al.* 1990; Ahmad *et al.* 1998; Noordam *et al.* 2009; Dawson 2013; Dawson and Markovich 2005). Despite the importance of sulfate in these physiological processes, little is known regarding sulfate homeostasis in humans nor is it routinely measured in clinical settings.

Given the potential importance of sulfate in the development of several disorders, we comprehensively characterized serum sulfate in 977 Amish individuals in order to (1) determine the heritability of serum sulfate, (2) examine the relationship between *SLC13A1* SNVs and serum sulfate, (3) identify additional novel genetic contributors through completion of the first exome-wide association study (ExWAS) and genome-wide association study (GWAS) of serum sulfate, and (4) investigate associations between sulfate-altering genotypes and relevant clinical phenotypes including aminotransferase levels, bone mineral density (BMD), and serum calcium.

## Materials and Methods

### Study population

This report is based on the Amish community living in Lancaster County, PA, whom our research group has been studying since 1993. This community was founded by several hundreds of individuals who immigrated to Lancaster County, PA from central Europe during the early 18th century, with the present-day Lancaster County Amish community comprised of their descendants (Lee *et al.* 2010). Cultural and religious beliefs have maintained the Amish as distinct from the general population. Due to the availability of extensive genealogical records (Agarwala *et al.* 2003), virtually all present-day Amish can be linked into a single, 14-generation pedigree. To date, we have screened ∼6500 Amish adults for a variety of risk factors related to cardiovascular disease (Mitchell *et al.* 2008), diabetes (Hsueh *et al.* 2000), and osteoporosis (Streeten *et al.* 2006) as part of the Amish Complex Disease Research Program (ACDRP).

Subjects included in this report are members of the Amish community in Lancaster County, PA who are at least 18 yrs old and have previously participated in one or more Institutional Review Board (IRB)-approved studies conducted at the University of Maryland School of Medicine Amish Research Clinic (ARC). Written informed consent was obtained from each participant.

### Genotyping

Exome-wide genotyping, including *SLC13A1* R12X (rs28364172), W48X (rs138275989), N174S (rs2140516), and R237C (rs139376972), was performed on 1725 Amish subjects using the Illumina Human Exome BeadChip (Illumina Inc., San Diego, CA). Genotyping was performed according to the manufacturer’s instructions and genotypes were assigned using Illumina GenomeStudio software (Illumina Inc.).

In subsequent follow-up investigations, genotyping of *SLC13A1* nonsense SNVs, R12X and W48X, was performed on additional Amish subjects using TaqMan single nucleotide polymorphism (SNP) genotyping assays (Life Technologies, Foster City, CA). In total, *SLC13A1* R12X and W48X genotypes were available in 3924 and 3782 unique individuals, respectively. For both SNPs, the TaqMan genotype concordance was > 99.8% in a subset of duplicate samples. For subjects genotyped using the Illumina Human Exome BeadChip and TaqMan SNP genotyping assays, genotype concordance between platforms was 100% for both *SLC13A1* R12X and W48X.

For 917 of the 977 subjects for which serum sulfate was measured, genome-wide genotyping was performed using the Affymetrix GeneChip Human Mapping 500K or 1M (version 6.0) arrays according to the manufacturer’s instructions (Affymetrix Inc., Santa Clara, CA). Genotype calls were performed using BRLMM (500K array) or Birdseed version 2 (1 M array). SNVs present on both arrays with an MAF > 1% were included in the analyses.

### Serum sulfate measurements

All known Amish carriers of the rare *SLC13A1* SNVs, R12X, W48X, and R237C, for whom fasting, serum aliquots were available, were selected for sulfate measurement. Such subjects consisted of participants from the Amish Pharmacogenomics of Antiplatelet Intervention (PAPI) Study (Shuldiner *et al.* 2009; Bozzi *et al.* 2015), the Amish Heredity and Phenotype Intervention (HAPI) Heart Study (Mitchell *et al.* 2008), the Amish Family Calcification Study (AFCS) (Post *et al.* 2007), the Amish Family Longevity Study (AFLS) (Mitchell *et al.* 2001; Sorkin *et al.* 2005), and the Amish Wellness Study (AWS) (Supplemental Material, File S1). All other subjects consisted of randomly selected participants of the Amish PAPI (Shuldiner *et al.* 2009; Bozzi *et al.* 2015) or HAPI Heart (Mitchell *et al.* 2008) studies for whom fasting serum aliquots were available.

Sulfate measurements were completed on frozen, fasting serum aliquots stored at −80^◦^. Sulfate concentration was determined by turbidimetry according to Dodgson and Price (1962) using a Quantichrom Sulfate Assay Kit (BioAssay Systems, Hayward, CA). In order to improve accuracy, a quadratic least squares fit was used instead of a linear fit to generate the standard curve (NIST/SEMATECH 2013). All standards and samples were measured in duplicate. For each sample, sulfate concentration was calculated as the mean of the duplicate measurements. Samples with an absolute difference > 20% were considered discordant duplicate measurements and were not included in the analysis. Of the 1003 samples in which serum sulfate was measured, 26 subjects’ samples were excluded due to discordant duplicate measurements, resulting in valid sulfate concentration measurements for 977 subjects’ samples.

### Serum alanine aminotransferase (ALT), aspartate aminotransferase (AST), calcium, and albumin measurements

Serum ALT, AST, calcium, and albumin measurements were collected as part of the Amish PAPI (Shuldiner *et al.* 2009; Bozzi *et al.* 2015) and Amish Family Longevity (Sorkin *et al.* 2005; Mitchell *et al.* 2001) studies and completed on fresh, fasting serum aliquots by Quest Diagnostics (Horsham, PA) at the time of subject enrollment.

### BMD measurements

BMD was measured as part of the Amish PAPI Study (Shuldiner *et al.* 2009; Bozzi *et al.* 2015) by dual-energy x-ray absorptiometry at the proximal femur, lumbar spine, one-third radius, and whole-body using a Hologic 4500W (Hologic). Daily phantom measurements were obtained to detect measurement shifts over time. Coefficients of variation for the repeat measures of the sites in normal volunteers were between 1.5% and 0.8%.

### Statistical analysis

The MAF for each nonsense SNV was obtained from the Total European Ancestry population from 1000 Genomes (1000g-EUR) and the European American population from the National Heart, Lung, and Blood Institute (NHLBI) Exome Sequencing Project (ESP) (ESP-EA). For each SNV, the total allele count was assumed to be 1338 in 1000g-EUR (*n* = 669) and 6728 in ESP-EA (*n* = 3364). For each nonsense SNV, the MAF in the Amish was compared to the MAF in 1000g-EUR and ESP-EA (when available) using a chi-squared test.

Association analyses between genotypes and serum sulfate, and other phenotypic measures, were conducted using a regression-based method that models variation of the trait of interest as a function of measured covariates, measured genotypes, and a polygenic component that accounts for phenotypic correlation due to relatedness. This method was implemented using the Mixed Models Analysis for Pedigrees and Populations (MMAP) program (O’Connell 2015). For each association analysis performed, individuals with a missing covariate, genotype, and/or trait of interest were excluded from the analysis. All analyses of serum sulfate included gender, study, and study-age as covariates. All analyses of clinical phenotypes included age and gender as covariates. All analyses of clinical phenotypes obtained in Amish PAPI Study (Shuldiner *et al.* 2009; Bozzi *et al.* 2015) participants containing serum sulfate as a covariate were limited to subjects for whom serum sulfate was measured using a serum aliquot collected at the time of participation in that study.

### Data availability

In accordance with our human subjects’ research protocols, we are unable to release the individual-level data set. We continue to deposit aggregate-level data into the National Institutes of Health (NIH) database of Genotypes and Phenotypes (dbGaP); however, given the Old Order Amish are a founder population with publically available pedigree data, depositing individual-level data on our subjects creates the potential for personal identification of our research subjects. In the past, the NIH has provided exemptions for this reason. Furthermore, all of our analyses were performed using the MMAP program (O’Connell 2015) which models variation of the trait of interest as a function of measured covariates, measured genotypes, and a polygenic component that accounts for phenotypic correlation due to relatedness. For this reason, outside investigators attempting to replicate our analyses will get different results if the family structure of the Old Order Amish is not accounted for in their analyses. We would be glad to assist any investigators who have questions or would like to perform related analyses regarding this data. We will certainly entertain requests for individual-level data and pedigree information with qualified investigators and collaborators after necessary modifications have been made to our study protocols. 

## Results

### SLC13A1 variants are enriched in the Amish

**Figure 1:**
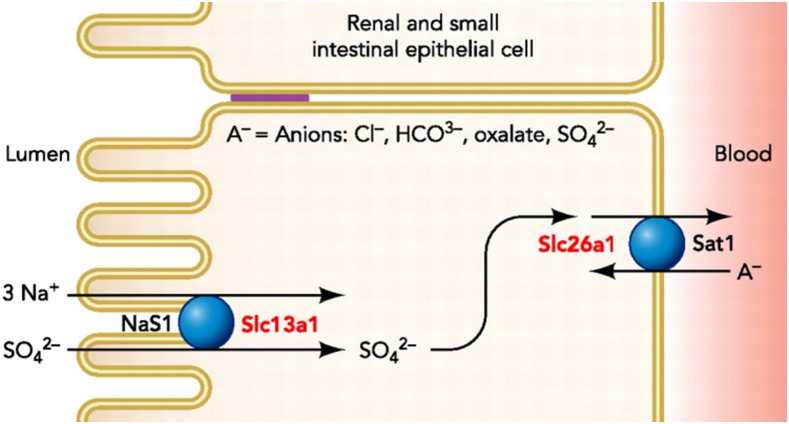
Sulfate transporters in the renal and small intestinal epithelial cell. Modified from Markovich (2012). Permission to reuse figure obtained from copyright holder.

In this Amish cohort (*n* = 1725), 43,459 of the >240,000 markers on the Illumina Human Exome BeadChip were polymorphic, 266 of which were nonsense SNVs. For 45 of the 266 nonsense SNVs, no MAF was reported in 1000g-EUR nor ESP-EA. For 131 of the 266 nonsense SNVs, the MAF was greater in this Amish cohort compared to the MAF in 1000g-EUR averaged with the MAF in ESP-EA. Of these 131 SNVs, 67 and 103 were significantly enriched (*P* < 0.05) compared to 1000G-EUR and ESP-EA, respectively (Table S1). With an intent to discover novel biology, we filtered out variants in genes with an associated Online Mendelian Inheritance in Man (OMIMa 2015) phenotype, leaving 118 nonsense SNVs. Two nonsense SNVs (c.34G > A, p.R12X and c.144C > T, p.W48X) in *SLC13A1* (Figure 1) were enriched 1.9-fold (0.43% *vs.* 0.23%) and 4.4-fold (0.87% *vs.* 0.20%), respectively, compared to ESP-EA allele frequencies. In addition, two missense SNVs (c.521T > C, p.N174S and c.877G > A, p.R237C) in *SLC13A1* were also present on the Illumina Exome BeadChip and enriched 1.2-fold (39% *vs.* 32%) and 10.7-fold (1.7% *vs.* 0.16%), respectively, compared to ESP-EA allele frequencies (Table S2).

Upon further genotyping of *SLC13A1* R12X and W48X in additional Amish subjects (n_R12X_ = 3924; n_W48X_ = 3782), the allele frequencies were enriched 1.2-fold (0.29% *vs.* 0.023%) and 3.7-fold (0.74% *vs.* 0.20%), respectively (Table S2). All four *SLC13A1* SNVs conformed to Hardy-Weinberg expectations (Table S2); no R12X homozygotes, W48X homozygotes, or R12X/W48X compound heterozygotes were observed. None of the four SNVs were in significant linkage disequilibrium (LD) with one another with the exception of N174S and R237C (*r*^2^ = 0.02 and |D’|=1.00, LOD ≥ 2) (Figure S1).

### SLC13A1 variants are associated with serum sulfate levels

Given the role of *SLC13A1* in sulfate (re)absorption, we measured sulfate levels in 977 individuals. Amish research subjects selected for sulfate measurement consisted of 481 males (49.2%) and 496 females (50.8%), with a mean age of 46.0 ± 14.4 yr, and a mean BMI of 27.1 ± 4.8 kg/m^2^ (Table 1). Serum sulfate was normally distributed in this population with an unadjusted mean of 0.36 mM (Figure S2). Serum sulfate was associated with increasing age (β = 0.0007, *P* = 4.9 × 10^−6^), but not with gender (β = −0.0049, *P* = 0.36). The total variance in serum sulfate explained by age, gender, and study was 13% (Table S3a). The residual heritability of serum sulfate after adjustment for age, gender, and study was 0.40 (*P* = 1.8 × 10^−16^).

**Table 1:** Characteristics of Old Order Amish research participants by cohort

Characteristic (Units)	Sulfate Cohort	PAPI Cohort	AFLS Cohort
Number (*n*)	977	684	264
Male (%)	49.2	49.8	47.3
Age ± SD (yr)	46.0 ± 14.4	45.0 ± 13.4	65.5 ± 10.4
BMI ± SD (kg/m^2^)	27.1 ± 4.8	27.1 ± 4.7	27.9 ± 5.2

PAPI, Pharmacogenomics of Antiplatelet Intervention; AFLS, Amish Family Longevity Study; SD, standard deviation; BMI, body mass index.

**Figure 2:**
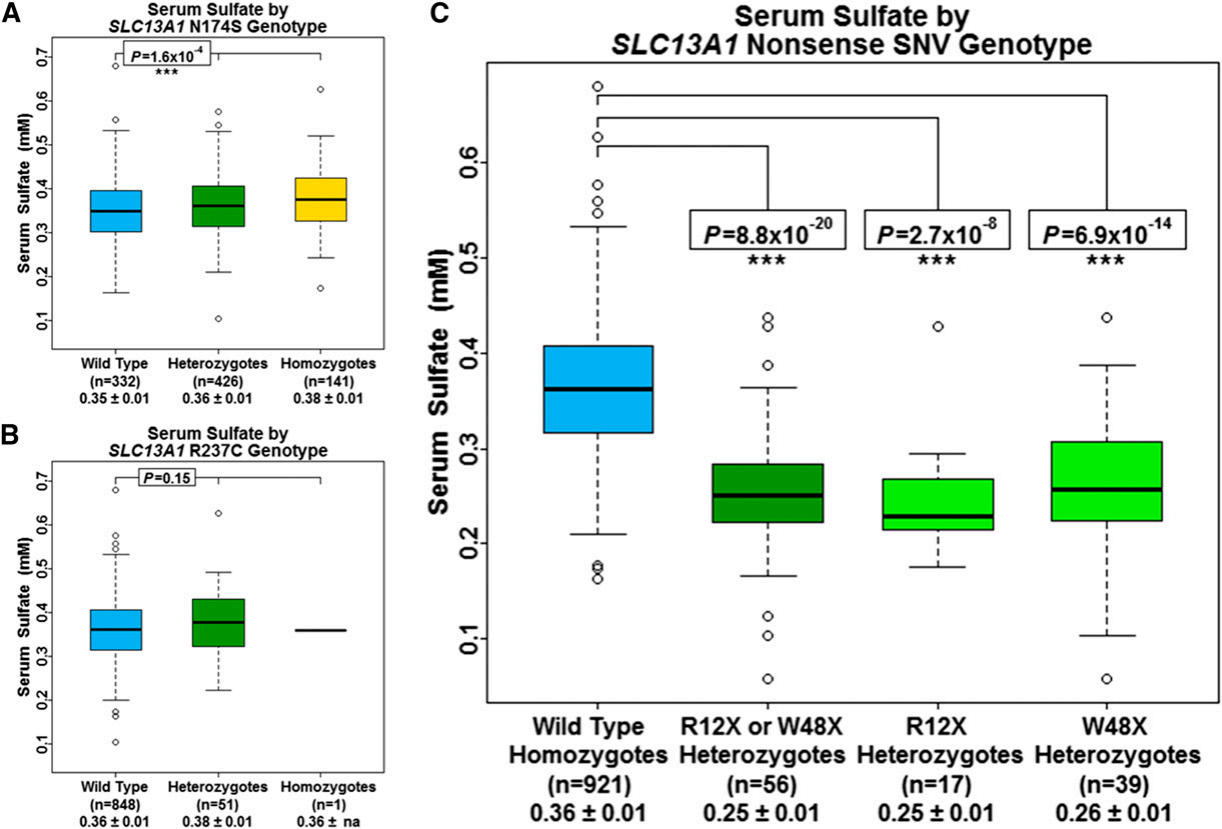
Serum sulfate concentration by *SLC13A1* genotype. Box and whisker plots: box represents 2nd and 3rd quartiles (IQR, interquartile range); horizontal band represents median value; whiskers represent 1st and 4th quartiles; ends of whiskers represent minimum and maximum values excluding outliers; open circles represent outliers (data point >1.5*IQR below the 1st quartile or above the 3rd quartile). Association analyses between genotypes and serum sulfate were conducted using a regression-based method that models variation of the trait of interest as a function of measured covariates, measured genotypes, and a polygenic component that accounts for phenotypic correlation due to relatedness. (A) Serum sulfate by *SLC13A1* N174S genotype. (B) Serum sulfate by *SLC13A1* R237C genotype. (C) Serum sulfate by *SLC13A1* nonsense SNV genotype. The *P*-value for the R12X or W48X heterozygotes (dark green box and whisker plot) results from the model including carrier status of an *SLC13A1* nonsense SNV (R12X or W48X) as a covariate. The *P*-values for the R12X heterozygotes and the W48X heterozygotes (light green box and whisker plots) result from the conditional model including both R12X and W48X as covariates.

In our primary model, we assessed associations between each of the four *SLC13A1* SNVs and serum sulfate independently (Table S3a–b). Specifically, we observed strong associations between R12X and W48X, and lower serum sulfate. *SLC13A1* R12X was associated with a 26.7% lower serum sulfate (β = −0.10 mM, *P* = 2.9 × 10^−7^) (Figure S3) and *SLC13A1* W48X was associated with a 26.5% lower serum sulfate (β = −0.10 mM, *P* = 1.3 × 10^−12^) (Figure S4). We evaluated the influence of N174S and R237C on serum sulfate levels in 900 of these individuals. In contrast to R12X and W48X, N174S was associated with a 3% higher serum sulfate per allele (β = 0.01 mM, *P* = 1.6 × 10^−4^) (Figure 2A). No association was observed between R237C and serum sulfate (*P* = 0.15) (Figure 2B). The estimated heritability after adjustment for each of these genotypes, along with the additive and total variance explained by each variant is shown in Table S3.

While LD data suggested that *SLC13A1* R12X and W48X were not correlated with each other, we performed conditional association analyses to evaluate whether these variants represented independent association signals (Table S2a). Indeed, both R12X and W48X were independently associated with a 27.6% (β = −0.10 mM, *P* = 2.7 × 10^−8^) and 27.3% (β = −0.10 mM, *P* = 6.9 × 10^−14^) decrease in serum sulfate, respectively (Figure 2C). *SLC13A1* R12X and W48X together, explained ∼31% of the additive variance and 9% of the total variance in serum sulfate. The increased significance of these two SNVs, along with the increase in additive and total variance explained, compared to our primary models, is consistent with these two loci being independent genetic determinants of serum sulfate.

Finally, we assessed the association between *SLC13A1* nonsense carriers and serum sulfate by including carrier status of an *SLC13A1* nonsense SNV (R12X or W48X) as a covariate (Table S3a). We observed a 27% decrease in serum sulfate among *SLC13A1* nonsense SNV carriers compared to subjects who do not carry R12X or W48X (β = −0.10 mM, *P* = 8.8 × 10^−20^) (Figure 2C). The estimated residual heritability after adjustment for *SLC13A1* nonsense SNV carrier status was 0.28 with carrier status of an *SLC13A1* nonsense SNV explaining 31% of the additive variance and 9% of the total variance in serum sulfate.

### SLC26A1 L348P is associated with serum sulfate levels

**Figure 3:**
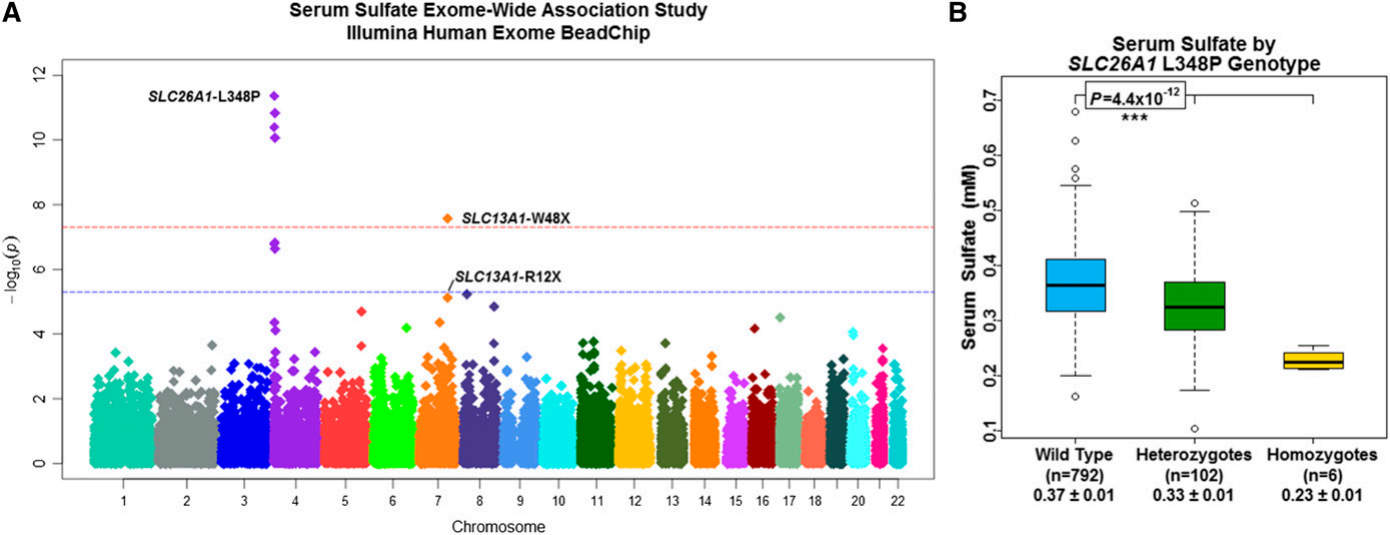
ExWAS of serum sulfate enables discovery of *SLC26A1* L348P. (A) Manhattan plot depicting association results (plotted as –log_10_ *P*-value on y-axis) of individual single nucleotide polymorphisms distributed across the 22 autosomes (x-axis) from the serum sulfate ExWAS performed using the Illumina Human Exome BeadChip platform (*n* = 900). Red horizontal dotted line indicates *P* = 5.0 × 10^−8^. Blue horizontal dotted line indicates *P =* 5.0 × 10^−6^. (B) Serum sulfate by *SLC26A1* L348P genotype. Box and whisker plots: box represents 2nd and 3rd quartiles (IQR, interquartile range); horizontal band represents median value; whiskers represent 1st and 4th quartiles; ends of whiskers represent minimum and maximum values, excluding outliers; open circles represent outliers (data point >1.5*IQR below the 1st quartile or above the 3rd quartile). Association analyses between genotypes and serum sulfate were conducted using a regression-based method that models variation of the trait of interest as a function of measured covariates, measured genotypes, and a polygenic component that accounts for phenotypic correlation due to relatedness.

An ExWAS of serum sulfate, performed using genotype data from the Illumina Human Exome BeadChip (*n* = 900) (Figure S5a), demonstrated genome-wide significance (*P* < 5.0 × 10^−8^) for W48X (β = −0.08 mM, *P* = 2.7 × 10^−8^), and suggestive-significance for R12X (β = −0.09 mM, *P* = 7.5 × 10^−6^) in *SLC13A1* on chromosome 7q31.32 (Figure 3 and Table S3b). The ExWAS also revealed a novel cluster of four SNVs spanning ∼3 megabases on chromosome 4p16.3 demonstrating genome-wide significance (Figure 3). These SNVs were found to be in strong LD with one another, *i.e.*, pairwise *r*^2^ = 0.49 to 0.97 and |D’|=0.79 to 1.00, LOD ≥ 2 (Figure S6), with L348P in *SLC26A1* (rs148386572; MAF = 0.06) being the most significant, associated with a 12% decrease in serum sulfate per allele (β = 0.05 mM, *P* = 4.4 × 10^−12^). *SLC26A1* encodes the basolateral membrane, sulfate-anion transporter 1 (Sat1), consistent with this locus as a true determinant of serum sulfate (Figure 1) and indicative of L348P causing decreased or loss-of-function in the Sat1 protein. *SLC26A1* L348P explained 3% of the additive variance and 5% of the total variance in serum sulfate (Table S3b). No other genomic region revealed association signals that met or exceeded genome-wide significance (Table S3).

A secondary ExWAS of serum sulfate, adjusting for *SLC13A1* R12X and W48X, and *SLC26A1* L348P genotypes, was also performed (*n* = 900) (Figure S5b and Table S5). No genomic region revealed association signals that met or exceeded genome-wide significance (Figure S7 and Table S4).

A GWAS of serum sulfate, performed using genotype data from the Affymetrix GeneChip (*n* = 917) (Figure S8), demonstrated genome-wide significance for a variant on chromosome 7 (rs17684427) and genome-wide suggestive-significance for a variant on chromosome 4 (rs362272) in strong LD with *SLC13A1* W48X and *SLC26A1* L348P, respectively (Figure S9, Figure S10, Figure S11, and Table S6). A secondary GWAS of serum sulfate, adjusting for rs17684427 and rs362272 genotypes, was also performed (*n* = 917) (Figure S12). No genomic region revealed association signals that met or exceeded genome-wide significance (Figure S13).

A model including all five SNV genotypes (*SLC13A1* R12X, W48X, N174S, and R237C, and *SLC26A1* L348P) was used to further estimate residual heritability (Table S3b). In this model, *SLC13A1* R12X, W48X, and N174S, and *SLC26A1* L348P were all independently associated with decreased serum sulfate while *SLC13A1* R237C was not. The estimated residual heritability after adjustment for *SLC13A1* R12X, W48X, N174S, and R237C, and *SLC26A1* L348P genotype was 0.31 with these variants, together, explaining ∼29% of the additive variance and 13% of the total variance in serum sulfate.

### SLC13A1 nonsense SNVs are associated with increased aminotransferase levels

**Figure 4:**
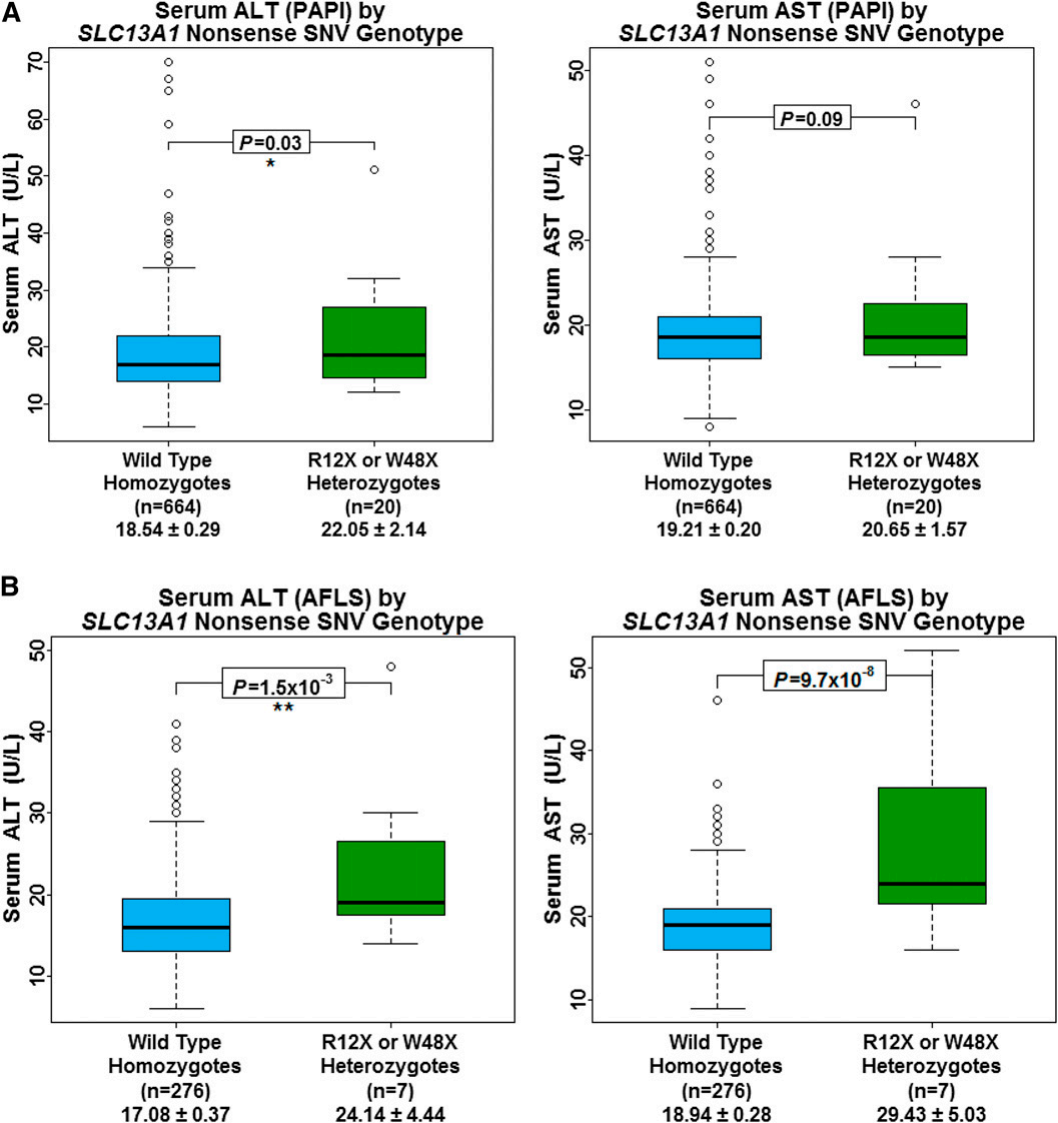
Serum alanine aminotransferase (ALT) and aspartate aminotransferase (AST) by *SLC13A1* nonsense SNV carrier status. Box and whisker plots: box represents 2nd and 3rd quartiles (IQR, interquartile range); horizontal band represents median value; whiskers represent 1st and 4th quartiles; ends of whiskers represent minimum and maximum values, excluding outliers; open circles represent outliers (data point >1.5*IQR below the 1st quartile or above the 3rd quartile). Association analyses between genotypes and serum sulfate were conducted using a regression-based method that models variation of the trait of interest as a function of measured covariates, measured genotypes, and a polygenic component that accounts for phenotypic correlation due to relatedness. (A) Amish PAPI Study subjects. (B) AFLS subjects.

Given sulfate’s role in drug metabolism, we assessed the association between *SLC13A1* nonsense SNV carriers and levels of alanine aminotransferase (ALT) and aspartate aminotransferase (AST) in 684 participants from the Amish PAPI Study (Shuldiner *et al.* 2009; Bozzi *et al.* 2015) (Table 1). ALT levels were 20% higher in *SLC13A1* nonsense SNV carriers compared to noncarriers (β = 3.7 U/L, *P* = 0.03). Similarly, a trending association was observed between *SLC13A1* nonsense carriers and increased AST (β = 2.1 U/L, *P* = 0.09) (Figure 4A). No association was observed between *SLC26A1* L348P, and ALT or AST levels (*P* = 0.92 and 0.37, respectively) (Table 2A).

**Table 2:** Associations between sulfate-lowering SNVs and clinical phenotypes

**(A)** Trait (PAPI)	Sulfate-lowering SNV(s)	*n*	Het.	Hom.	β_SNV_ ± SE (U/L)	β_SNV_/Mean_WT_	*P*_SNV_
ALT	*SLC13A1* R12X or W48X	684	20	0	3.73 ± 1.74	0.20	**0.03**
	*SLC26A1* L348P	650	71	6	−0.08 ± 0.84	0.00	0.92
AST	*SLC13A1* R12X or W48X	684	20	0	2.13 ± 1.26	0.11	0.09
**(B)** Trait (AFLS)	SNV Covariate(s)	*n*	Het.	Hom.	β_SNV_ ± SE (U/L)	β_SNV_/Mean_WT_	*P*_SNV_
ALT	*SLC13A1* R12X or W48X	264	7	0	7.25 ± 2.33	0.42	**2.0 × 10^−3^**
	*SLC26A1* L348P	232	21	2	0.81 ± 1.20	0.04	0.50
AST	*SLC13A1* R12X or W48X	264	7	0	10.42 ± 1.92	0.55	**1.3 × 10^−7^**
	*SLC26A1* L348P	232	21	2	2.42 ± 0.89	0.13	**6.8 × 10^−3^**
**(C)** Trait	Sulfate-lowering SNV(s)	*n*	Het.	Hom.	β_SNV_ ± SE	β_SNV_/Mean_WT_	*P*_SNV_
Whole-body BMD (g/cm^2^)	*SLC13A1* R12X or W48X	679	20	0	−0.03 ± 0.02	−0.03	0.19
	*SLC26A1* L348P	645	69	6	−0.03 ± 0.01	−0.03	**6.8 × 10^−3^**
Calcium (mg/dl)	*SLC13A1* R12X or W48X	684	20	0	0.00 ± 0.07	0.00	0.98
	*SLC26A1* L348P	650	71	6	0.11 ± 0.03	0.01	**1.4 × 10^−3^**
Corrected calcium (mg/dl)	*SLC13A1* R12X or W48X	684	20	0	−0.03 ± 0.06	0.00	0.68
	*SLC26A1* L348P	650	71	0	0.06 ± 0.03	0.01	0.06
**(D)** BMD Trait	Sulfate-lowering SNV(s)	*n*	Het.	Hom.	β_SNV_ ± SE (g/cm^2^)	β_SNV_/Mean_WT_	*P*_SNV_
Midarm	*SLC13A1* R12X or W48X	680	20	0	−0.02 ± 0.01	−0.04	0.08
	*SLC26A1* L348P	646	70	6	−0.01 ± 0.01	−0.02	**0.03**
1/3-arm	*SLC13A1* R12X or W48X	680	20	0	−0.03 ± 0.02	−0.04	0.08
	*SLC26A1* L348P	646	70	6	−0.02 ± 0.01	−0.02	**0.03**
Total-arm	*SLC13A1* R12X or W48X	680	20	0	−0.02 ± 0.01	−0.04	0.06
	*SLC26A1* L348P	646	70	6	−0.02 ± 0.01	−0.02	**0.01**
Ultradistal-arm	*SLC13A1* R12X or W48X	680	20	0	−0.02 ± 0.01	−0.03	0.26
	*SLC26A1* L348P	646	70	6	−0.01 ± 0.01	−0.03	**0.03**
Femoral neck	*SLC13A1* R12X or W48X	681	20	0	−0.02 ± 0.03	−0.02	0.46
	*SLC26A1* L348P	647	70	6	−0.02 ± 0.01	−0.02	0.26
Intertrochanter	*SLC13A1* R12X or W48X	680	20	0	−0.04 ± 0.04	−0.03	0.35
	*SLC26A1* L348P	646	70	6	−0.05 ± 0.02	−0.04	**5.1 × 10^−3^**
Total-hip	*SLC13A1* R12X or W48X	681	20	0	−0.03 ± 0.03	−0.03	0.28
	*SLC26A1* L348P	647	70	6	−0.04 ± 0.01	−0.04	**0.01**
Trochanter	*SLC13A1* R12X or W48X	680	20	0	−0.03 ± 0.03	−0.04	0.23
	*SLC26A1* L348P	646	70	6	−0.03 ± 0.01	−0.03	**0.04**
Total-spine	*SLC13A1* R12X or W48X	679	20	0	−0.03 ± 0.03	−0.03	0.39
	*SLC26A1* L348P	645	70	6	−0.03 ± 0.02	−0.03	0.10

Adjusted for age and gender. The *P*-value of significant associations are highlighted in bold text. (A) Serum alanine aminotransferase (ALT) and aspartate aminotransferase (AST) levels from the Amish Pharmacogenomics of Antiplatelet Intervention (PAPI) Study. (B) Serum ALT and AST levels from the Amish Family Longevity Study (AFLS). (C) Whole-body bone mineral density (BMD) and serum calcium from the Amish PAPI Study. (D) Additional BMD measurements from the Amish PAPI Study. Het., heterozygotes; Hom., homozygotes; SE, standard error; WT, wild type.

We were able to replicate these associations in an independent Amish cohort consisting of 264 participants from the AFLS (Mitchell *et al.* 2001; Sorkin *et al.* 2005) who were, on average, 20.5 yr older than the 684 participants from the Amish PAPI Study (Shuldiner *et al.* 2009; Bozzi *et al.* 2015) (Table 1). ALT and AST levels were found to be 42% and 55% higher, respectively, in *SLC13A1* nonsense SNV carriers than in noncarriers (β = 7.3 U/L, *P* = 2.0 × 10^−3^ and β = 10.4 U/L, *P* = 1.3 × 10^−7^, respectively) (Figure 4B). Additionally, an association between *SLC26A1* L348P and a 13% increase in AST per allele was observed in this cohort (β = 2.4 U/L, *P* = 6.8 × 10^−3^) (Table 2B).

### SLC26A1 sulfate-lowering SNVs are associated with altered serum calcium and BMD

**Figure 5:**
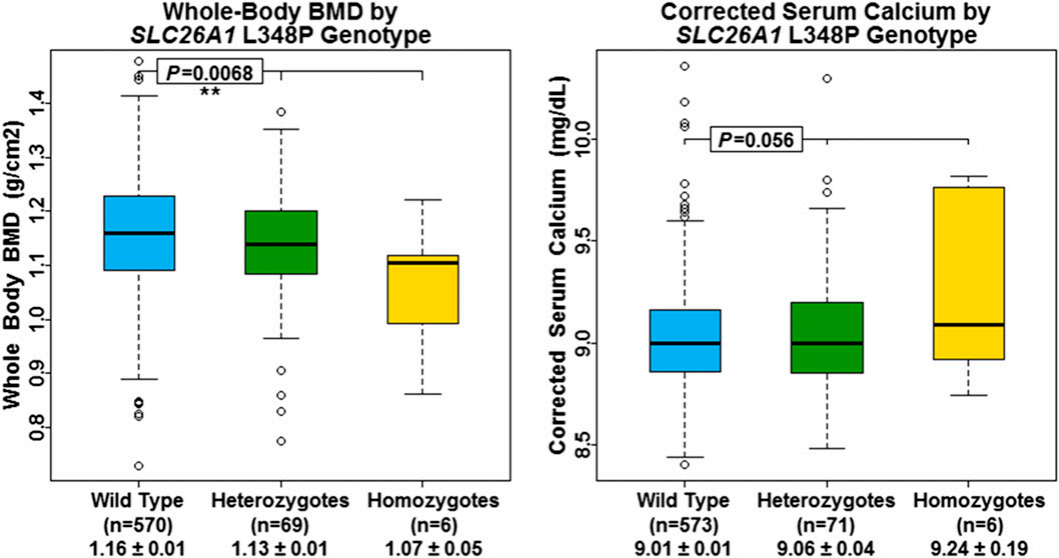
Whole-body BMD and corrected serum calcium by *SLC26A1* L348P genotype. Box and whisker plots: box represents 2nd and 3rd quartiles (IQR, interquartile range); horizontal band represents median value; whiskers represent 1st and 4th quartiles; ends of whiskers represent minimum and maximum values, excluding outliers; open circles represent outliers (data point >1.5*IQR below the 1st quartile or above the 3rd quartile). Association analyses between genotypes and serum sulfate were conducted using a regression-based method that models variation of the trait of interest as a function of measured covariates, measured genotypes, and a polygenic component that accounts for phenotypic correlation due to relatedness.

Given the role of sulfate in cartilage and bone development, we assessed the association between *SLC13A1* nonsense SNV carriers and whole-body BMD. No association was observed between *SLC13A1* nonsense carriers and whole-body BMD (*P* = 0.19); however, *SLC26A1* L348P was significantly associated with decreased whole-body BMD (β = −0.03 g/cm^2^, *P* = 6.8 × 10^−3^) (Figure 5 and Table 2C). Upon further investigation, *SLC26A1* L348P was also associated with several other BMD measurements while *SLC13A1* nonsense SNV carrier status was not (Table 2D and Table S7). Furthermore, *SLC26A1* L348P was significantly associated with increased total serum calcium (β = 0.11 mg/dL, *P* = 1.4 × 10^−3^), and showed a nearly significant association with albumin-adjusted serum calcium (β = 0.06, *P* = 0.06) (Figure 5 and Table 2C). No association was observed between *SLC13A1* nonsense SNV carriers, and serum calcium nor albumin-adjusted serum calcium (*P* = 0.98 and 0.68, respectively) (Table 2C).

### Clinical phenotypes are not associated with serum sulfate concentration

Serum sulfate concentration by itself was not associated with any of the aforementioned clinical phenotypes with the exception of trochanter and intertrochanter BMD. Lower serum sulfate was marginally associated with lower trochanter (β = 0.13 g/cm^2^, *P* = 0.03) and lower intertrochanter BMD (β = 0.18 g/cm^2^, *P* = 0.04) (Table S8).

## Discussion

Using genomic approaches to unveil new human biology, we systematically identified nonsense SNVs that are rare in the general population but enriched in the Amish due to founder effect. Two nonsense SNVs (R12X and W48X), as well as two missense SNVs (N174S and R237C), in *SLC13A1* were not only enriched in the Amish but likely functional, thus we hypothesized that these SNVs would be associated with altered serum sulfate due to altered sulfate (re)absorption, and perhaps other traits for which sulfate may play a role. We further conducted the first ExWAS and GWAS of serum sulfate in an attempt to identify other genetic contributors of serum sulfate, a trait not measured clinically despite its important physiological role in human development, disease, and drug toxicity. To the best of our knowledge, our group is the first to establish that serum sulfate is heritable (h^2^ = 0.40, *P =* 1.8 × 10^−16^). In addition, these studies have identified novel associations between R12X, W48X, and N174S in *SLC13A1*, and L348P in *SLC26A1*, and serum sulfate levels. Further clinical characterization of the three sulfate-lowering variants implicates sulfate-wasting in liver function, and perhaps BMD and calcium homeostasis.

In regards to *SLC13A1* R12X, our findings are consistent with work performed by Bowling *et al.*, which demonstrated an association between R12X and increased urinary fractional excretion index (FEI) of sulfate in a cohort with ASD (Bowling *et al.* 2013). Furthermore, the R12X allele has been shown by Lee *et al.* to abolish 100% of NaS1 sulfate transport in *Xenopus* oocytes (Lee *et al.* 2006). It is likely that this complete loss of NaS1 function occurs through a nonsense-mediated decay (NMD) mechanism. While *SLC13A1* W48X has not been described in the literature to date, the similar effect size of R12X and W48X on serum sulfate and their lack of LD with one another suggest similar loss-of-functionality. Together, these two SNVs alone explain ∼31% of the additive variance and 9% of the total variance of serum sulfate in the Amish. Based on previous experience, these estimates are likely to be lower in outbred populations. Nevertheless, these estimates clearly validate our approach of systematically identifying and studying nonsense SNVs to glean insights into human biology.

In addition, we observed an association between the more common *SLC13A1* N174S variant and increased serum sulfate, albeit an effect size of ∼1/10th the magnitude of *SLC13A1* R12X or W48X, consistent with gain-of-function in NaS1. In contrast, Bowling *et al.* found N174S to be associated with increased FEI of sulfate (Bowling *et al.* 2013) in a cohort with ASD while Lee *et al.* found the N174S allele to abolish > 60% of NaS1 sulfate transport (Lee *et al.* 2006). With the exception of R12X, other loss-of-function variants, such as W48X, were not accounted for by Bowling *et al.*

Our ExWAS and GWAS findings support the results of our candidate gene study regarding the sulfate-lowering effect of R12X and W48X in *SLC13A1*, while also identifying L348P in *SLC26A1* as an additional genetic contributor of serum sulfate. *SLC26A1* encodes Sat1, the basolateral membrane, sulfate-anion transporter, consistent with this locus as a true determinant of serum sulfate. While *SLC26A1* L348P has not been described in the literature to our knowledge, at half the effect size of *SLC13A1* R12X or W48X, each copy of the L348P allele results in an additive decrease in serum sulfate. This association is suggestive of *SLC26A1* L348P causing decreased or loss-of-function in the Sat1 protein; however functional studies are needed to fully understand the extent to which this occurs. It should be noted that all three of the sulfate-lowering SNVs identified in this report are enriched in the Amish, with *SLC13A1* W48X and *SLC26A1* L348P being markedly enriched. Therefore, our study is likely better powered to detect these variants compared to other ExWAS or GWAS of serum sulfate in outbred populations of comparable size.

Lastly, our results indicate that serum sulfate is a heritable trait, and while our results explain a large portion of the genetic component of serum sulfate, there is an equally large portion yet to be explained. It is possible that much of this heritability can be explained by additional variants in *SLC13A1*, *SLC26A1*, and/or other sulfate transporters that were missed by the genotyping arrays used in this study. Such variants have the potential to be identified through other genotyping arrays, exome sequencing, and/or copy number analysis, thus studies aiming to identify additional genetic variants in genes associated with serum sulfate in the Amish, as well as outbred populations, are warranted.

### SLC13A1 and aminotransferase levels

Serum AST and ALT are biomarkers of hepatocyte integrity and thus elevations in these serum aminotransferases suggest liver cell injury (Giannini *et al.* 2005; Gowda *et al.* 2009). Given sulfate’s role in drug metabolism and increased sensitivity to acetaminophen-induced hepatotoxicity exhibited by *Slc13a1*- and *Slc26a1*-knockout mice (Lee *et al.* 2006; Dawson *et al.* 2010; Markovich 2012), we hypothesized that individuals with sulfate-lowering variants would have higher liver aminotransferase levels. The association between *SLC13A1* nonsense SNV carriers and higher aminotransferase levels in participants of the Amish PAPI Study (Shuldiner *et al.* 2009; Bozzi *et al.* 2015) supports our hypothesis, with even greater effects seen in participants of the AFLS (Mitchell *et al.* 2001; Sorkin *et al.* 2005). The increase in aminotransferase levels seen in *SLC13A1* nonsense SNV carriers is consistent with increased susceptibility to drugs or exogenous substrates metabolized through sulfation. This is potentially due to decreased inward transport of sulfate into cells, independent of serum sulfate concentration, resulting in decreased ability to detoxify xenobiotics. In contrast to the Amish PAPI Study (Shuldiner *et al.* 2009; Bozzi *et al.* 2015), participants of the AFLS (Mitchell *et al.* 2001; Sorkin *et al.* 2005) were not asked to discontinue prescription medications and/or supplements 7 d prior to their initial clinic visit (File S1). We attribute the increased effect of sulfate-lowering variants in participants of the AFLS to routine medication/supplement use, as well as increased participant age which may render hepatocytes more susceptible to damage from xenobiotics. This phenomenon may partially explain the lack of an association between *SLC26A1* L348P and elevated aminotransferase levels in the participants of the younger Amish PAPI Study (Shuldiner *et al.* 2009; Bozzi *et al.* 2015), along with differential tissue expression of *SLC13A1* and *SLC26A1*, and/or L348P causing decreased, but sufficient, Sat1 function, as opposed to a complete loss-of-function. Moreover, the associations observed between sulfate-lowering SNVs and aminotransferase level in this study may be underestimated given the relative drug-naïve lifestyle practice by the Amish community. Additional studies are warranted to better understand the importance of sulfate in human physiology and its potential role in disease and drug toxicity.

### SLC26A1, and BMD and serum calcium

Sulfate’s physiological role in bone and cartilage is well known. Sulfated proteoglycans are a critical component of extracellular matrices in cells throughout the body, specifically in connective tissues, and are required for maintaining normal bone and cartilage structure (Yanagishita 1993; Fernandes *et al.* 1997). The essentiality of sulfate for proper bone and cartilage formation is demonstrated by the spectrum of osteochondrodysplasias in humans with homozygous, loss-of-function mutations in *SLC26A2* (Hastbacka *et al.* 1996; Hastbacka *et al.* 1999; OMIMb 2015), as well as dog and sheep with homozygous loss-of-function mutations in *Slc13a1* (Neff *et al.* 2012; Zhao *et al.* 2012). We thus hypothesized that individuals with sulfate-lowering SNVs would have altered BMD. We observed several significant associations between *SLC26A1* L348P and decreased BMD measurements, but no association between BMD and *SLC13A1* nonsense carrier status, nor serum sulfate concentration. However, compared to *SLC26A1* L348P (allele count ∼70, 6 homozygotes), there was far less power to detect such associations with *SLC13A1* nonsense SNVs in this study due to the lower allele frequency (allele count = 20) and the lack of homozygotes. The lack of an association with *SLC13A1* nonsense variants may also be explained by differential tissue expression of *SLC13A1* and *SLC26A1*.

The association between increased serum calcium and *SLC26A1* L348P may be specific to decreased Sat1 (*SLC26A1*) function and is seemingly independent of serum sulfate. These findings are consistent with mouse models in which hyposulfatemic, *Slc26a1*-knockout mice exhibit nephrocalcinosis and increased calcium oxalate kidney stone formation while hyposulfatemic, *Slc13a1*-knockout mice do not exhibit these manifestations (Dawson *et al.* 2010; Markovich 2011a,b, 2012). Additionally, *SLC26A1* has been suggested to play a role in humans with recurrent calcium oxalate kidney stones (Dawson *et al.* 2013). While the mechanism for nephrocalcinosis, calcium oxalate urolithiasis, and increased serum calcium is not totally clear, these phenotypes associate with decreased Sat1 function, suggesting they may be specific to anion transport rather than sulfate transport. We hypothesize that the *SLC26A1* L348P allele decreases Sat1 function, decreasing sulfate transport into the blood as well as decreasing anion transport into cells. As a result, serum oxalate and thus urine oxalate concentrations may increase resulting in the formation of calcium oxalate crystals which either accumulate to form stones and/or increase serum calcium levels. We do not have an adequate number of subjects with renal stones to address this hypothesis further.

### Conclusions

Sulfate is an essential micronutrient, as impaired sulfation capacity has been reported in a number of human conditions including reduced xenobiotic clearance, skeletal dysplasias, premature pubarche, ASD, and neurological disease (Alberti *et al.* 1999; Bowling *et al.* 2013; Heafield *et al.* 1990; Ahmad *et al.* 1998; Noordam *et al.* 2009; Dawson 2013; Dawson and Markovich 2005). Despite sulfate’s known role in the biotransformation of hormones, neurotransmitters, glycosaminoglycans (GAGs), cerebrosides, and xenobiotics (Dawson 2011; Markovich 2011a, 2014; Council 2005), sulfate is not routinely measured clinically (Dawson 2011, 2013). In this study, we measured and comprehensively characterized serum sulfate in 977 Amish subjects. We are the first to estimate the heritability of serum sulfate and the first to perform an ExWAS and a GWAS of serum sulfate. We identified novel associations between serum sulfate and three SNVs in *SLC13A1*, which encodes the apical membrane, sodium-sulfate cotransporter, NaS1. Through our ExWAS of serum sulfate, we further identified a novel association with L348P in *SLC26A1*, which encodes the basolateral membrane, sulfate-anion transporter, Sat1. We also investigated associations between sulfate-lowering SNVs, aminotransferase levels, BMD, and serum calcium, results of which implicate sulfate and these SNVs in important human metabolism, physiology, and disease processes.

In conclusion, this study demonstrates the power and translational potential of leveraging genetic data in founder populations like the Old Order Amish to unveil and better understand the phenotypic consequences of rare, deleterious variants enriched by genetic drift. Further, our clinical findings warrant additional studies to better understand the importance of sulfate in human physiology, disease, and drug toxicity.

## Supplementary Material

Supplemental Material
