# Supplementary material for: From Genotype to Phenotype: Nonsense Variants in SLC13A1 Are Associated with Decreased Serum Sulfate and Increased Serum Aminotransferases
Source: G3 (Bethesda). 2016 Jul 13;6(9):2909–18. doi: 10.1534/g3.116.032979 (PMC5015947; doi:10.1534/g3.116.032979)
Supplement: Supplemental Material [file supp_6_9_2909__index.html]

From Genotype to Phenotype: Nonsense Variants in SLC13A1 Are Associated with Decreased Serum Sulfate and Increased Serum Aminotransferases — Supplemental Material 

# From Genotype to Phenotype: Nonsense Variants in *SLC13A1* Are Associated with Decreased Serum Sulfate and Increased Serum Aminotransferases

## Supplemental Material for Tise *et al.*, 2016

**Files in this Data Supplement:**

- File S1 - Supplementary methods. (.pdf, 231 KB)
- Table S1 - Minor allele frequency enrichment summary of nonsense variants in the Old Order Amish (n=1,725) compared to 1000g(EUR) and ESP(EA) populations. (.pdf, 86 KB)
- Table S2 - SNV characteristics in relevant cohorts. (.pdf, 23 KB)
- Table S3 - Associations between serum sulfate and *SLC13A1* nonsense SNVs (n=977). (.pdf, 119 KB)
- Table S4 - Top 20 serum sulfate exome-wide association study (ExWAS) results using the Illumina Human Exome BeadChip platform (n=900). (.pdf, 137 KB)
- Table S5 - Top 20 serum sulfate exome-wide association study (ExWAS) results, adjusted for *SLC13A1* R12X and W48X, and *SLC26A1* L348P, using the Illumina Human Exome BeadChip platform (n=900). (.pdf, 137 KB)
- Table S6 - Top 20 serum sulfate genome-wide association study (GWAS) results using Affymetrix GeneChip platform (n=917). (.pdf, 135 KB)
- Table S7 - Associations between sulfate-lowering SNVs, and bone mineral density (BMD) measurements. (.pdf, 144 KB)
- Table S8 - Associations between serum sulfate, and serum alanine aminotransferase (ALT) and aspartate aminotransferase (AST) levels and bone mineral density (BMD) measurements. (.pdf, 116 KB)
- Figure S1 - Linkage disequilibrium (LD) plots depicting r2 and D' amongst *SLC13A1* SNVs within the entire Amish cohort (n=1,725). (.tif, 172 KB)
- Figure S2 - Descriptive statistics and distribution of serum sulfate in 977 Old Order Amish research participants. (.tif, 137 KB)
- Figure S3 - Serum sulfate by *SLC13A1* R12X genotype. (.tif, 12 KB)
- Figure S4 - Serum sulfate by *SLC13A1* W48X genotype. (.tif, 13 KB)
- Figure S5 - QQ plot for the serum sulfate ExWAS performed using the Illumina Human Exome BeadChip platform (n=900). (.tif, 258 KB)
- Figure S6 - Linkage disequilibrium plots depicting r2 and D' amongst the top serum sulfate ExWAS hits on chromosome 4 from the Illumina Human Exome BeadChip platform (n=900). (.tif, 497 KB)
- Figure S7 - Serum sulfate ExWAS results adjusted for *SLC13A1* R12X, *SLC13A1* W48X, and *SLC26A1* L348P (n=900). (.tif, 259 KB)
- Figure S8 - QQ plot for serum sulfate GWAS using Affymetrix GeneChip platform (n=917). (.tif, 38 KB)
- Figure S9 - Serum sulfate GWAS results (n=917). (.tif, 116 KB)
- Figure S10 - Linkage disequilibrium plots depicting r2 and Dï¿½ amongst *SLC26A1*ï¿½ L348P (from the Illumina Human Exome BeadChip platform) and the top serum sulfate GWAS hits on chromosome 7 from the Affymetrix GeneChip platform (n=879, subject overlap between Illumina Human Exome BeadChip and Affymetrix GeneChip platforms). (.tif, 424 KB)
- Figure S11 - Linkage disequilibrium plots depicting r2 and Dï¿½ amongst *SLC13A1*ï¿½ W48X (from the Illumina Human Exome BeadChip platform) and the top serum sulfate GWAS hits on chromosome 4 from the Affymetrix GeneChip platform (n=879, subject overlap between Illumina Human Exome BeadChip and Affymetrix GeneChip platforms). (.tif, 320 KB)
- Figure S12 - QQ plot for serum sulfate GWAS adjusted for rs17684427 and rs362272 using Affymetrix GeneChip platform (n=917). (.tif, 38 KB)
- Figure S13 - Serum sulfate GWAS results adjusted for rs17684427 and rs362272 (n=917). (.tif, 117 KB)
