# Supplementary material for: From Genotype to Phenotype: Nonsense Variants in SLC13A1 Are Associated with Decreased Serum Sulfate and Increased Serum Aminotransferases
Source: G3 (Bethesda). 2016 Jul 13;6(9):2909–18. doi: 10.1534/g3.116.032979 (PMC5015947; doi:10.1534/g3.116.032979)
Supplement: Supplemental Material [file supp_g3.116.032979_FileS1.pdf]

Study Populations

Protocols for the studies described below were approved by the Institutional Review Board at the University of Maryland School of Medicine. All participants gave written informed consent.

Summaries

The Amish Pharmacogenomics of Antiplatelet Intervention (PAPI) Study [1]: The Amish PAPI Study was initiated in 2006 to identify genetic variants that influence the efficacy of oral antiplatelet agents (e.g. aspirin and clopidogrel). In addition to a detailed medical exam including blood pressure, anthropometry, and a standard fasting lipid panel, subjects underwent an 8-day clopidogrel treatment with platelet aggregation studies pre- and posttreatment and 1 hour after ingestion of 324 mg aspirin on day 8.

Amish Family Longevity Study (AFLS) [2, 3]: The AFLS was established in 2001 to identify genes that influence longevity and longevity-related traits in humans. Long-lived Amish probands (age > 90) were identified using the Amish Genealogical Database and through surveys sent to all Amish households listed in the Amish Church Directory [4] and recruited to the study along with all willing offspring and spouses of offspring. Phenotypic characterization included a detailed medical exam including blood pressure, anthropometry, and a standard fasting lipid panel.

The Amish Heredity and Phenotype Intervention (HAPI) Heart Study [5]: The Amish HAPI Heart Study began in 2003 to identify genes that interact with environmental exposures to influence risk for cardiovascular disease. Subjects underwent a detailed medical examination including blood pressure, anthropometry, a standard fasting lipid panel and four short-term interventions designed to challenge cardiovascular function including blood pressure responses to (1) the cold pressor stress test and (2) a high salt diet, (3) triglyceride excursion in response to a high fat challenge, and (4) response in platelet aggregation to aspirin therapy.

Amish Family Calcification Study (AFCS) [6]: The AFCS was initiated in 2001 to identify the determinants of vascular calcification and to evaluate the relationship between calcification of bone and vascular tissue. Subjects underwent a detailed medical examination including blood pressure, anthropometry, a standard fasting lipid panel, and the measurement of coronary artery calcification (CAC) by electron bean computed tomography (EBCT).

Amish Wellness Study (AWS): Through the Amish Research Clinic and the Amish Wellness Mobile, recruitment into the Amish Complex Disease and Wellness Biobank is ongoing. Accruing at a rate of approximately 1,500 subjects per year, we expect to complete total ascertainment of virtually all Amish adults in Lancaster County (approximately 12,000 subjects) within the next 4 years.

Detailed Descriptions from Cited Articles

<u>The Amish Pharmacogenomics of Anti-Platelet Intervention (PAPI) Study [1]</u>

The Amish Pharmacogenomics of Antiplatelet Intervention (PAPI) Study. Subjects were recruited between August 2006 and October 2008. Subjects were Old Order Amish aged ≥ 20 years who were generally healthy based upon exclusion criteria for major illnesses including kidney, liver and symptomatic cardiac disease. Prescription medications, vitamins and supplements were discontinued for 1 week prior to the initial study visit. Medical and family histories, anthropometry, and physical examinations were conducted at the Amish Research Clinic in Lancaster, PA. The 429 Amish PAPI subjects comprised a number of relative pairs informative for estimating heritabilities, including 105 parent-offspring pairs, 175 sib pairs, 1 grandparent-grandchild pairs, 48 avuncular pairs, and 12 first cousin pairs.

Blood samples were obtained following an overnight fast. Complete blood count with platelet number and serum lipids (total cholesterol, high-density lipoprotein cholesterol (HDL-C) and triglycerides) were assayed by Quest Diagnostics (Horsham, PA). All subjects had triglyceride <400 mg/dL and low-density lipoprotein (LDL-C) levels were calculated using the Friedewald equation. Hyperlipidemia was defined as an LDL-C of greater than 160 mg/dL and/or use of prescription cholesterol-lowering medications. Hypertension was defined as systolic blood pressure ≥140 mm Hg, and/or diastolic blood pressure ≥90 mm Hg, and/or use of prescription blood pressure lowering medications. Diabetes was defined as a self-reported history. Current smoking status included self-reported use of cigarette, pipe, or cigar.

After baseline platelet aggregation measurements were made, subjects were given an oral

loading dose of 300 mg clopidogrel followed by 75 mg per day for 6 days. Follow-up platelet aggregation studies were repeated one hour following the last dose of clopidogrel. A second follow-up platelet aggregation measurement was made on the same day one hour after oral ingestion of 324 mg of chewable aspirin (4 x 81 mg). Platelet function was assessed by optical aggregometry. Briefly, venous blood was drawn into 3.2% citrate-anticoagulated tubes (Becton-Dickinson, Franklin Lakes, NJ). Platelet rich plasma was prepared from whole blood and platelet counts adjusted to 200,000 platelets/μL with platelet poor plasma. Platelet aggregation was assessed in a PAP8E Aggregometer (Bio/Data Corporation, Horsham, PA) after stimulating samples with ADP (2, 5, 10, and 20 μmol/L) or arachidonic acid (1.6 mmol/L).

Amish Family Longevity Study (AFLS) [2, 3]

The Old Order Amish of Lancaster County, PA is a founder population that our group has been studying since 1993. The AFLS was established in 2001 to identify genes that influence longevity and longevity-related traits in humans [3]. Long-lived Amish probands (age > 90) were identified using the Amish Genealogical Database and through surveys sent to all Amish households listed in the Amish Church Directory [4], and recruited to the study along with all willing offspring and spouses of offspring.

The Amish Heredity and Phenotype Intervention (HAPI) Heart Study [5]

Participants for the HAPI Heart Study were recruited from the Amish community of Lancaster County, PA. Only subjects aged 20 years and older were eligible to participate and age-eligible family members of all participating subjects were encouraged to enroll. Before enrollment, potential study participants were visited at home by a study nurse and Amish liaison

team, who performed a screening examination to determine eligibility. Although most subjects qualified for all interventions, there were separate exclusion criteria for each to enable subjects to participate in only those interventions for which they qualified. A summary of the global exclusion criteria and intervention specific exclusion criteria is provided in Table I.

A total of 1003 individuals were identified for recruitment and received home visits to establish eligibility. Of these, 78 (7.8%) refused to participate and 45 (4.5%) did not meet one or more of the global eligibility criteria. Twelve eligible and consented individuals withdrew before completing any of the interventions, leaving a final sample size of 868 participants. The study protocol required a 4-week period to complete all 4 interventions. After obtaining permission from their prescribing physicians, all medications, vitamins, and supplements were discontinued for 7 days before Clinic Visit 1 and for the duration of the study. As Amish do not drive cars, transportation to the Amish Research Clinic (Lancaster, PA) was provided. Subjects were instructed to fast for 12 hours before their appointment, to abstain from excessive physical activity on the morning of the

Table I. Exclusion criteria for HAPI Heart Study

Study-wide exclusion criteria:

- Age< 20 yrs
- Non-Amish descent
- Currently pregnant or postpartum <6 m
- Blood pressure at the time of screening >180/105 (SBB/DBP) mm Hg
- Prescription medication use potentially affecting outcomes and vitamin or over-the-counter remedies that cannot be willingly or safely discontinued from 1 week before protocol initiation and until the end of the study (ie, β-blockers; calcium channel antagonists; ACE inhibitors; diuretics; lipid-lowering agents; nitrates; systemic glucocorticoids; adrenergic or cholinergic-acting agents, including cold formulas and antidepressants; and diet-weight loss agents)
- Coexisting malignancy
- Serum creatinine>2.0 mg/dL
- AST or ALT >twice the upper limit of normal
- Hematocrit <32%
- TSH <0.4 or >5.5 mIU/L

Intervention-specific exclusion criteria:

- Cold pressor stress test: history of Raynaud's disease
- High-fat challenge: malabsorption disorders, lactose intolerance, symptoms of gallbladder disease, and/or history of pancreatitis
- Dietary salt intervention: stage III or greater congestive heart failure and/or allergies to foods in the diet
- Aspirin intervention: history of bleeding disorder, gastrointestinal bleeding, blood pressure at the time of screening >160/95 mm Hg, current use of aspirin for a condition that would place the subject at increased risk if it were to be discontinued for 14 days before protocol initiation (eg, history of unstable angina, myocardial infarction, angioplasty, coronary artery bypass grafting, atrial fibrillation, stroke or transient ischemic attack, type 2 diabetes, or deep vein thrombosis/other thrombosis), polycythemia (hematocrit >52%), thrombocytosis (platelet count > 500000), thrombocytopenia (platelet count <75000), surgery within the last 6 months, aspirin allergy, current breastfeeding, and/or aggregation with collagen 5 μg/mL<6.65 Ω or >26 Ω or no aggregation at baseline with arachidonic acid

ACE, Angiotensin-converting enzyme; *ALT*, alanine aminotransferase; *AST*, aspartate aminotransferase; *TSH*, thyroid-stimulating hormone.

visit, and to bring a first morning void urine sample. Clinic Visit 1 lasted for 8 to 10 hours, during which time the following were performed: physical examination, urine pregnancy test, fasting blood sample for pre-aspirin analyses of platelet aggregation and inflammatory markers, blood pressure monitoring, CPT, high fat challenge, electrocardiogram, ultrasound measurement of carotid intima media thickness, measurement of pulse wave velocity and ankle-brachial index (ABI), and echocardiogram. All blood samples were obtained through an indwelling angiocatheter.

The CPT was conducted by having the subjects immerse their right hand and wrist in ice water for a period of 2½ minutes. Before immersion, blood pressure measurements were taken every 5 minutes for at least 20 minutes. Nine additional blood pressure measurements were taken during and after the cold pressor stimulus at minutes 1, 2, 3, 4, 5, 7.5, 10, 15, and 20 after immersion. A brachial artery reactivity test was performed during the course of the cold pressor stimulus to measure changes in brachial artery diameter during and after the CPT stimulus. The high-fat challenge was administered 1 hour after completion of the CPT. Before the beginning of the fat challenge, blood was drawn to measure fasting lipids and brachial artery flow-mediated vasodilation was measured to assess fasting endothelial function.

The high-fat challenge, prepared in the form of a whipping cream milk shake, was standardized to consist of 782 calories per m^2 of body surface with 77.6% of calories from fat, 19.2% from carbohydrate, and 3.1% from protein. After ingestion, blood was drawn at 1, 2, 3, 4, and 6 hours to assess the triglyceride excursion. Brachial artery flow-mediated vasodilations were also performed at 2, 4, and 6 hours after the fat challenge to assess post-prandial effects on endothelial function. The subject rested and remained fasting during the 6 hours post-fat challenge.

The aspirin intervention was begun the day after Clinic Visit 1; for the next 14 consecutive days the subject took 81 mg aspirin every day. One to 3 days before the second clinic visit a nurse and liaison performed a home visit to ensure compliance. On the 14th day, the subjects took their aspirin shortly before arriving at the Amish Research Clinic for Clinic Visit 2. Fasting blood was drawn to measure post-aspirin platelet aggregation. The subjects also collected and brought their first morning urine sample for measurement of 11-dehydrothromboxane B2. To assess compliance, a pill count was performed and each subject kept a diary. The subjects were permitted to miss up to 4 aspirin doses over the 2-week period and still be included in the final analysis, provided that they took the aspirin for at least 3 consecutive days before Clinic Visit 2. The aspirin intervention could be extended for up to 3 days (17 days total) to meet this criterion.

The Monday after Clinic Visit 1, subjects began 6 days of a high-salt diet, followed by a 6- to 14-day washout period, and then 6 days of a low-salt diet. The 6-day course for the high-/lowsalt interventions was chosen so that the special diet would not interfere with their Sunday church meal. All meals were culturally appropriate and prepared by an Amish caterer and provided to the subjects by home delivery. The high- and lowsalt diets contained 280 and 40 meq sodium per day, respectively. Both diets contained 140 meq potassium per day. The diets provided approximately 2900 kcal/d with 55% from carbohydrates, 15% from protein, and 30% from fats. On the sixth day of each diet, the subjects wore an ambulatory blood pressure monitor to assess their blood pressure during a 24-hour period. A nurse and Amish liaison visited the subjects on the third and fifth days of each diet to ensure that they were not adversely affected by the diets and to check their food diary for compliance. In addition, the subjects were instructed to collect the first morning urine sample on days 4, 5, and 6 for measurement of sodium and

creatinine as an independent assessment of compliance to the salt diets. The 3-day means of the urine sodium/creatinine ratios were 162.1 ± 60.8 meq/mg (range 20.4-444.1) and 33.5 ± 18.6 meq/mg (range 6.9-164.9) for the high- and low-salt diets, respectively, indicating a N4-fold decrease in urinary sodium excretion from high- to low-salt diets. During the high- and low-salt interventions, subjects also recorded into their diaries the times they went to bed and woke up to define active and inactive periods. Direct measurements of physical activity were obtained during this period by having subjects wear accelerometers (Actical, Mini Mitter/Respironics, Bend, OR) for 6 consecutive days.

The Family Amish Calcification Study (AFCS) [6]

The Amish Family Calcification Study was initiated in 2001 to identify the determinants of vascular calcification and to evaluate the relationship between calcification of bone and vascular tissue among members of the Old Order Amish community in Lancaster County. Subjects were initially recruited into the AFCS on the basis of their participation in an earlier family study of bone mineral density, although recruitment guidelines were later modified to allow other interested individuals in the community to participate. All first- and second-degree relatives of these new participants also were invited to participate in the AFCS. Recruitment efforts were made without regard to CVD health status.

In the AFCS coronary artery calcification was measured in men aged 30 years and older or women aged 40 years or older. All AFCS participants underwent a detailed clinical examination at the Amish Research Clinic in Strasburg, PA, including assessment of potential risk factors for CVD and a medical history interview. Examinations were conducted after an overnight fast. Height and weight were measured with a stadiometer and calibrated scale with shoes removed and in light

clothing. Body mass index (kg/m^2) was calculated. Systolic (first phase) blood pressure (BP) and diastolic (fifth phase) BP were obtained in triplicate with a standard sphygmomanometer with the subject sitting for at least 5 minutes. For these analyses, BP was defined as the mean of the second and third measurements. Pulse pressure was defined as the difference between the systolic and diastolic BPs. Medication lists were obtained at the participant's home by a study nurse. Smoking habits were recorded by questionnaire; subjects were classified as current smokers or not.

Blood samples were obtained for determination of fasting glucose and lipid levels. Glucose concentrations were assayed with a Beckman glucose analyzer using the glucose oxidase method. Lipid concentrations were assayed by Quest Diagnostics (Baltimore, Md). Low-density lipoprotein cholesterol levels were calculated using the Friedewald equation. Diabetes mellitus was defined as a fasting glucose ≥126 mg/dL or use of diabetes medications; impaired fasting glucose was defined as glucose ≥100 mg/dL.

References

1. Shuldiner AR, O'Connell JR, Bliden KP, Gandhi A, Ryan K, Horenstein RB, Damcott CM, Pakyz R, Tantry US, Gibson Q, et al: **Association of cytochrome P450 2C19 genotype with the antiplatelet effect and clinical efficacy of clopidogrel therapy.** *Jama* 2009, **302:**849-857.

2. Mitchell BD, Hsueh WC, King TM, Pollin TI, Sorkin J, Agarwala R, Schaffer AA, Shuldiner AR: **Heritability of life span in the Old Order Amish.** *Am J Med Genet* 2001, **102:**346-352.

3. Sorkin J, Post W, Pollin TI, O'Connell JR, Mitchell BD, Shuldiner AR: **Exploring the genetics of longevity in the Old Order Amish.** *Mech Ageing Dev* 2005, **126:**347-350.

4. *Church directory of the Lancaster County Amish.* Gordonville, PA: Pequea Pub.; 1996.

5. Mitchell BD, McArdle PF, Shen H, Rampersaud E, Pollin TI, Bielak LF, Jaquish C, Douglas JA, Roy-Gagnon MH, Sack P, et al: **The genetic response to short-term interventions affecting cardiovascular function: rationale and design of the Heredity and Phenotype Intervention (HAPI) Heart Study.** *Am Heart J* 2008, **155:**823-828.

6. Post W, Bielak LF, Ryan KA, Cheng YC, Shen H, Rumberger JA, Sheedy PF, 2nd, Shuldiner AR, Peyser PA, Mitchell BD: **Determinants of coronary artery and aortic calcification in the Old Order Amish.** *Circulation* 2007, **115:**717-724.
