# Supplementary material for: From Genotype to Phenotype: Nonsense Variants in SLC13A1 Are Associated with Decreased Serum Sulfate and Increased Serum Aminotransferases
Source: G3 (Bethesda). 2016 Jul 13;6(9):2909–18. doi: 10.1534/g3.116.032979 (PMC5015947; doi:10.1534/g3.116.032979)
Supplement: Supplemental Material [file supp_g3.116.032979_TableS1.pdf]

Table S1. Minor allele frequency enrichment summary of nonsense variants in the Old Order Amish (n=1,725) compared to 1000g$_{(EUR)}$ and ESP$_{(EA)}$ populations.

1000g$_{(EUR)}$

	Ratio >1	Ratio <1	Ratio Unknown	Total
P **<0.05**	67	79	0	146
P **>0.05**	30	32	0	62
P **N.C.**	34	4	20	58
**Total**	131	115	20	266

ESP$_{(EA)}$

	Ratio >1	Ratio <1	Ratio Unknown	Total
P **<0.05**	103	84	0	187
P **>0.05**	22	23	0	45
P **N.C.**	6	8	20	34
**Total**	131	115	20	266

Abbreviations: 1000g(EUR), Total European Ancestry population from 1000 Genomes; ESP(EA), European American population from the National Heart, Lung, and Blood Institute (NHLBI) Exome Sequencing Project (ESP); Ratio, minor allele frequency in the Old Order Amish divided by the minor allele frequency in the relevant population; N.C., not calculated.
