# Supplementary material for: From Genotype to Phenotype: Nonsense Variants in SLC13A1 Are Associated with Decreased Serum Sulfate and Increased Serum Aminotransferases
Source: G3 (Bethesda). 2016 Jul 13;6(9):2909–18. doi: 10.1534/g3.116.032979 (PMC5015947; doi:10.1534/g3.116.032979)
Supplement: Supplemental Material [file supp_g3.116.032979_TableS2.pdf]

Table S2. SNV characteristics in relevant cohorts.

Cohort	Allele Characteristics	*SLC13A1* rs28364172 (R12X)		*SLC13A1* rs138275989 (W48X)		*SLC13A1* rs2140516, (N174S)		*SLC13A1* rs139376972 (R237C)		*SLC26A1* rs148386572 (L348P)	
		**G**	**A**	**C**	**T**	**T**	**C**	**G**	**A**	**A**	**G**
**1000g(EUR)**	**Allele Freq. (%)**	99.60	0.40	99.90	0.10	66.30	33.70	n/a	n/a	99.70	0.30
**ESP(EA)**	**Allele Freq. (%)**	99.77	0.23	99.80	0.20	68.49	31.51	99.84	0.16	99.78	0.22
**Old Order Amish**	**Allele Freq. (%)**	99.71	0.29	99.26	0.74	60.61	39.39	98.26	1.74	93.19	6.81
	**Homozygotes**	3901	0	3726	0	621	255	1666	1	1503	13
	**Heterozygotes**	23		56		848		58		209	
	***P*HWE**	0.85		0.65		0.21		0.55		0.08	
	**Total**	3924		3782		1724		1725		1725	
**Sulfate Cohort**	**Allele Freq. (%)**	99.13	0.87	98.00	2.00	60.67	39.33	97.06	2.94	93.67	6.33
	**Homozygotes**	960	0	938	0	333	141	848	1	792	6
	**Heterozygotes**	17		39		426		51		102	
	***P*HWE**	0.78		0.52		0.81		0.80		0.18	
	**Total**	977		977		900		900		900	

Abbreviations: 1000g(EUR), Total European Ancestry population from 1000 Genomes; ESP(EA), European American population from the National Heart, Lung, and Blood Institute (NHLBI) Exome Sequencing Project (ESP); Freq., frequency; HWE, Hardy-Weinberg Equilibrium.
