# Supplementary material for: From Genotype to Phenotype: Nonsense Variants in SLC13A1 Are Associated with Decreased Serum Sulfate and Increased Serum Aminotransferases
Source: G3 (Bethesda). 2016 Jul 13;6(9):2909–18. doi: 10.1534/g3.116.032979 (PMC5015947; doi:10.1534/g3.116.032979)
Supplement: Supplemental Material [file supp_g3.116.032979_TableS3.pdf]

Table S3a. Associations between serum sulfate and *SLC13A1* nonsense SNVs (n=977).

SNV covariate(s)	β_{SNV} ± SE (mM)	β_{SNV}/ $Mean_{WT}$	P_{SNV}	h^2 ± SE	P_{h2}	%AVE	R^2	%TVE
**No SNVs**	-	-	-	0.40 ± 0.07	1.8x10^{-16}	-	0.13	-
**R12X**	-0.096 ± 0.019	-0.27	2.9x10^{-7}	0.40 ± 0.07	7.5x10^{-15}	1	0.15	2
**W48X**	-0.096 ± 0.013	-0.27	1.3x10^{-12}	0.31 ± 0.07	6.0x10^{-9}	24	0.19	6
**R12X**<br>**W48X**	-0.099 ± 0.018<br>-0.099 ± 0.013	-	2.7x10^{-8}<br>6.9x10^{-14}	0.28 ± 0.07	5.3x10^{-6}	31	0.22	9
**Nonsense Carrier**	-0.099 ± 0.011	-0.27	8.8x10^{-20}	0.28 ± 0.07	5.3x10^{-6}	31	0.22	9

SLC13A1 R12X and W48X genotyping was performed for all individuals for whom serum sulfate was measured. Analyses were adjusted for gender, study, and study-age. Abbreviations: SE, standard error; h^2, heritability; AVE, additive variance explained; TVE, total variance explained.

Table S3b. Associations between serum sulfate, and *SLC13A1* and *SLC26A1* SNVs (n=900).

SNV covariate(s)	β_{SNV} ± SE (mM)	β_{SNV}/ $Mean_{WT}$	P_{SNV}	h^2 ± SE	P_{h2}	%AVE	R^2	%TVE
No SNVs	-	-	-	0.44 ± 0.08	7.9x10^{-17}	-	0.10	-
N174S	0.012 ± 0.003	0.03	1.6x10^{-4}	0.43 ± 0.08	3.6x10^{-16}	2	0.12	2
R237C	0.015 ± 0.010	0.04	0.15	0.44 ± 0.08	1.6x10^{-16}	0	0.11	0
R12X<br>(GWAS)	-0.088 ± 0.019	-	7.5x10^{-6}	0.42 ± 0.08	2.2x10^{-14}	3	0.13	2
W48X<br>(GWAS)	-0.082 ± 0.015	-	2.7x10^{-8}	0.36 ± 0.08	1.6x10^{-10}	18	0.15	5
L348P<br>(GWAS)	-0.046 ± 0.006	0.12	4.4x10^{-12}	0.43 ± 0.08	2.1x10^{-15}	3	0.15	5
R12X<br>W48X<br>L348P	-0.094 ± 0.018<br>-0.080 ± 0.014<br>-0.045 ± 0.006	-	3.5x10^{-7}<br>9.5x10^{-9}<br>9.7x10^{-13}	0.32 ± 0.08	2.9x10^{-7}	27	0.22	12
R12X<br>W48X<br>N174S<br>R237C<br>L348P	-0.091 ± 0.018<br>-0.076 ± 0.014<br>0.009 ± 0.003<br>0.008 ± 0.010<br>-0.045 ± 0.006	-	5.8x10^{-7}<br>5.3x10^{-8}<br>3.4x10^{-3}<br>0.40<br>9.2x10^{-13}	0.31 ± 0.08	6.4x10^{-7}	29	0.23	13

SLC13A1 N174S and R237C, and *SLC26A1* L348P genotyping was performed on 900 of the 977 individuals for whom serum sulfate was measured. Analyses were adjusted for gender, study, and study-age. Abbreviations: SE, standard error; h^2, heritability; AVE, additive variance explained; TVE, total variance explained.
