# Supplementary material for: From Genotype to Phenotype: Nonsense Variants in SLC13A1 Are Associated with Decreased Serum Sulfate and Increased Serum Aminotransferases
Source: G3 (Bethesda). 2016 Jul 13;6(9):2909–18. doi: 10.1534/g3.116.032979 (PMC5015947; doi:10.1534/g3.116.032979)
Supplement: Supplemental Material [file supp_g3.116.032979_TableS4.pdf]

Table S4. Top 20 serum sulfate exome-wide association study (ExWAS) results using the Illumina Human Exome BeadChip platform (n=900).

rs Number	Gene	Type	AA Change	Chrom.	Position	Freq. ExWAS	P_{HWE}	Freq. $1000g_{(EUR)}$	Freq. $ESP_{(EA)}$	Enrich. $1000g_{(EUR)}$	Enrich. $ESP_{(EA)}$	β_{SNV}	P_{SNV}
rs148386572	*SLC26A1*	exonic	L348P	4p16.3	983,684	0.063	0.21	0.003	0.002	21.1	28.4	-0.046	**4.4E-12**
rs59932476	*DOK7*	exonic	G248R	4p16.3	3,491,504	0.079	0.15	0.034	0.032	2.3	2.5	-0.040	1.5E-11
rs141442388	*PIGG*	exonic	A434T	4p16.3	517,332	0.064	0.10	0.001	-	64.4	-	-0.043	4.0E-11
rs11549516	*LRPAP1*	exonic	R113H	4p16.3	3,526,645	0.081	0.20	0.036	0.035	2.3	2.3	-0.038	8.6E-11
rs138275989	*SLC13A1*	exonic	W48X	7q31.32	122,821,111	0.017	1.00	0.001	0.002	16.7	8.4	-0.082	**2.7E-08**
rs2498323	*HGFAC*	exonic	R644Q	4p16.3	3,451,109	0.098	0.24	0.082	0.100	1.2	1.0	-0.028	1.5E-07
rs2306242	*GAK*	exonic	K1167R	4p16.3	843,720	0.105	0.29	0.037	0.038	2.9	2.8	-0.028	1.6E-07
rs362272	*HTT*	exonic	V2786I	4p16.3	3,234,980	0.247	0.35	0.309	0.296	0.8	0.8	-0.019	2.3E-07
rs962040	*SGCZ, TUSC3*	intergenic	-	8p22	15,311,877	0.251	0.83	0.733	-	0.3	-	-0.017	5.8E-06
rs28364172	*SLC13A1*	exonic	R12X	7q31.32	122,839,967	0.008	1.00	0.004	0.002	2.1	3.6	-0.088	**7.5E-06**
rs117725783	*TMEM71*	exonic	D78G	8q24.22	133,764,112	0.027	0.70	0.015	0.020	1.8	1.3	0.043	1.4E-05
rs2082412	*UBLCP1, IL12B*	intergenic	-	5q33.3	158,717,789	0.166	0.21	0.226	-	0.7	-	0.019	2.0E-05
rs3213094	*IL12B*	intronic	-	5q33.3	158,750,769	0.166	0.21	0.223	-	0.7	-	0.019	2.0E-05
rs8069166	*FAM101B*	exonic	-	17p13.3	293,138	0.023	0.58	0.024	0.033	1.0	0.7	0.047	3.1E-05
rs7685686	*HTT*	intronic	-	4p16.3	3,207,142	0.344	0.11	0.433	-	0.8	-	-0.014	4.3E-05
rs117360089	*DMTF1*	exonic	A8P	7q21.12	86,800,364	0.024	0.55	0.021	0.016	1.1	1.5	0.045	4.4E-05
rs362331	*HTT*	exonic	Y2309H	4p16.3	3,215,835	0.343	0.11	0.433	0.420	0.8	0.8	-0.014	4.4E-05
rs4647930	*FGFRL1*	exonic	P362Q	4p16.3	1,018,705	0.243	0.02	0.267	0.270	0.9	0.9	-0.016	4.5E-05
rs202020297	*HIVEP2*	exonic	-	6q24.2	143,095,100	0.020	0.84	-	0.001	-	13.9	-0.050	6.5E-05
rs13336804	*CIITA*	exonic	V782A	16p13.13	11,001,694	0.119	0.96	0.094	0.084	1.3	1.4	-0.020	6.8E-05

Abbreviations: Freq. ExWAS, allele frequency in the 900 Amish subjects included in the ExWAS; Freq. $1000g_{(EUR)}$, allele frequency in Total European Ancestry population from 1000 Genomes; Freq. $ESP_{(EA)}$, allele frequency in European American population from the National Heart, Lung, and Blood Institute (NHLBI) Exome Sequencing Project (ESP); Enrich. $1000g_{(EUR)}$, enrichment of allele frequency in the 900 Amish subjects included in the ExWAS compared to allele frequency in Total European Ancestry population from 1000 Genomes (Freq. ExWAS/Freq. $1000g_{(EUR)}$); Enrich. $ESP_{(EA)}$, enrichment of allele frequency in the 900 Amish subjects included in the ExWAS compared to allele frequency in European American population from the NHLBI ESP (Freq. ExWAS/Freq. $ESP_{(EA)}$).
