# Supplementary material for: From Genotype to Phenotype: Nonsense Variants in SLC13A1 Are Associated with Decreased Serum Sulfate and Increased Serum Aminotransferases
Source: G3 (Bethesda). 2016 Jul 13;6(9):2909–18. doi: 10.1534/g3.116.032979 (PMC5015947; doi:10.1534/g3.116.032979)
Supplement: Supplemental Material [file supp_g3.116.032979_TableS5.pdf]

Table S5. Top 20 serum sulfate exome-wide association study (ExWAS) results, adjusted for *SLC13A1* R12X and W48X, and *SLC26A1* L348P, using the Illumina Human Exome BeadChip platform (n=900).

rs Number	Gene	Type	AA Change	Chrom.	Position	Freq. ExWAS	P_{HWE}	Freq. $1000g_{(EUR)}$	Freq. $ESP_{(EA)}$	Enrich. $1000g_{(EUR)}$	Enrich. $ESP_{(EA)}$	β_{SNV}	P_{SNV}
rs117725783	*TMEM71*	exonic	D78G	8q24.22	133,764,112	0.027	0.70	0.015	0.020	1.8	1.3	0.040	1.8E-05
rs7797400	*SEMA3C, LOC100128317*	intergenic	-	7q21.11	80,812,398	0.238	0.07	0.371	-	0.6	-	-0.015	1.9E-05
rs3213094	*IL12B*	intronic	-	5q33.3	158,750,769	0.166	0.21	0.223	-	0.7	-	0.017	4.4E-05
rs2082412	*UBLCP1, IL12B*	intergenic	-	5q33.3	158,717,789	0.166	0.21	0.226	-	0.7	-	0.017	4.4E-05
rs962040	*SGCZ, TUSC3*	intergenic	-	8p22	15,311,877	0.251	0.83	0.733	-	0.3	-	-0.015	4.4E-05
rs117360089	*DMTF1*	exonic	A8P	7q21.12	86,800,364	0.024	0.55	0.021	0.016	1.1	1.5	0.041	4.7E-05
rs2282192	*C9ORF156*	exonic	V324M	9q22.33	100,672,338	0.273	0.64	0.270	0.271	1.0	1.0	0.014	9.3E-05
rs8069166	*FAM101B*	exonic	-	17p13.3	293,138	0.023	0.58	0.024	0.033	1.0	0.7	0.041	1.1E-04
rs1129411	*SP110*	exonic	W112R	2q37.1	231,077,725	0.127	0.28	0.883	0.883	0.1	0.1	0.018	1.1E-04
rs2069556	*TG*	exonic	D1312G	8q24.22	133,920,518	0.354	0.38	0.609	0.615	0.6	0.6	-0.013	1.3E-04
rs10076782	*RNF145*	intronic	-	5q33.3	158,604,963	0.326	0.03	0.264	-	1.2	-	0.012	1.9E-04
rs1473247	*RNF145*	intronic	-	5q33.3	158,603,571	0.326	0.03	0.264	-	1.2	-	0.012	1.9E-04
rs789560	*RAB3IP, LINC01481*	intergenic	-	12q15	70,331,827	0.179	0.86	0.120	-	1.5	-	0.015	2.1E-04
rs6493352	*MTMR10*	exonic	R648H	15q13.3	31,234,064	0.130	0.01	0.188	0.168	0.7	0.8	-0.017	2.4E-04
rs61743026	*OR8H3*	exonic	T254I	11q12.1	55,890,609	0.061	0.01	0.094	0.081	0.6	0.8	-0.025	2.6E-04
rs140206966	*OR8I2*	exonic	T278K	11q12.1	55,861,616	0.061	0.01	.	0.000	-	130.1	-0.025	2.6E-04
rs61888286	*OR8H1*	exonic	S214F	11q12.1	56,057,898	0.061	0.01	0.092	0.080	0.7	0.8	-0.025	2.6E-04
rs193240312	*RFXAP*	exonic	M137T	13q13.3	37,393,904	0.037	0.16	0.020	0.018	1.9	2.1	0.028	3.4E-04
rs6033098	*LOC339593, LINC00687*	intergenic	-	20p12.2	11,289,485	0.057	0.95	0.049	-	1.2	-	0.023	3.6E-04
rs187821820	*SPEN*	exonic	S3466G	1p36.13	16,264,027	0.030	0.83	-	0.000	-	128.8	-0.032	3.7E-04

Abbreviations: Freq. ExWAS, allele frequency in the 900 Amish subjects included in the ExWAS; Freq. $1000g_{(EUR)}$, allele frequency in Total European Ancestry population from 1000 Genomes; Freq. $ESP_{(EA)}$, allele frequency in European American population from the National Heart, Lung, and Blood Institute (NHLBI) Exome Sequencing Project (ESP); Enrich. $1000g_{(EUR)}$, enrichment of allele frequency in the 900 Amish subjects included in the ExWAS compared to allele frequency in Total European Ancestry population from 1000 Genomes (Freq. ExWAS/Freq. $1000g_{(EUR)}$); Enrich. $ESP_{(EA)}$, enrichment of allele frequency in the 900 Amish subjects included in the ExWAS compared to allele frequency in European American population from the NHLBI ESP (Freq. ExWAS/Freq. $ESP_{(EA)}$).
