# Supplementary material for: From Genotype to Phenotype: Nonsense Variants in SLC13A1 Are Associated with Decreased Serum Sulfate and Increased Serum Aminotransferases
Source: G3 (Bethesda). 2016 Jul 13;6(9):2909–18. doi: 10.1534/g3.116.032979 (PMC5015947; doi:10.1534/g3.116.032979)
Supplement: Supplemental Material [file supp_g3.116.032979_TableS6.pdf]

Table S6. Top 20 serum sulfate genome-wide association study (GWAS) results using Affymetrix GeneChip platform (n=917).

rs Number	Gene	Type	AA Change	Chrom.	Position	Freq. GWAS	P_{HWE}	Freq. $1000g_{(EUR)}$	Freq. $ESP_{(EA)}$	Enrich. $1000g_{(EUR)}$	Enrich. $ESP_{(EA)}$	β_{SNV}	P_{SNV}
rs17684427	*CADPS2*	intronic	-	7q31.32	122,482,977	0.017	1.00	0.023	-	0.7	-	-0.086	9.4E-10
rs16864709	*P2RY1, RAP2B*	intergenic	-	3q25.2	152,636,304	0.002	1.00	-	-	-	-	0.191	2.7E-07
rs362272	*HTT*	exonic	V2786I	4p16.3	3,234,980	0.232	0.39	0.309	0.296	0.8	0.8	-0.020	3.0E-07
rs6690583	*MCOLN2, MCOLN3*	intergenic	-	1p22.3	85,466,337	0.157	0.23	0.788	-	0.2	-	-0.022	2.2E-06
rs6859018	*UBLCP1, IL12B*	intergenic	-	5q33.3	158,736,992	0.165	0.31	0.224	-	0.7	-	0.021	3.1E-06
rs3212217	*IL12B*	intronic	-	5q33.3	158,755,130	0.164	0.08	0.230	-	0.7	-	0.020	3.8E-06
rs2082412	*UBLCP1, IL12B*	intergenic	-	5q33.3	158,717,789	0.165	0.15	0.226	-	0.7	-	0.020	4.0E-06
rs10486023	*FOXP2*	intronic	-	7q31.1	114,178,653	0.016	1.00	0.075	-	0.2	-	-0.067	4.3E-06
rs3213094	*IL12B*	intronic	-	5q33.3	158,750,769	0.165	0.11	0.223	-	0.7	-	0.020	4.9E-06
rs16982644	*FAM49A*	intronic	-	2p24.2	16,829,430	0.025	0.48	0.043	-	0.6	-	-0.048	1.0E-05
rs1371143	*MCOLN2*	intronic	-	1p22.3	85,455,532	0.145	0.08	0.160	-	0.9	-	-0.021	1.1E-05
rs4338946	*ADAM23*	intronic	-	2q33.3	207,317,096	0.001	1.00	0.078	-	0.0	-	0.278	1.2E-05
rs16890309	*ZFAND3*	intronic	-	6p21.2	37,973,613	0.001	1.00	-	-	-	-	0.278	1.2E-05
rs11041824	*LMO1*	intronic	-	11p15.4	8,270,351	0.063	0.09	0.090	-	0.7	-	-0.030	1.6E-05
rs17617539	*TSPAN12, ING3*	intergenic	-	7q31.31	120,588,565	0.022	0.62	0.084	-	0.3	-	-0.050	1.9E-05
rs7795223	*CDK14*	intronic	-	7q21.13	90,290,392	0.178	0.53	0.245	-	0.7	-	0.018	2.0E-05
rs7522239	*MCOLN3*	UTR3	-	1p22.3	85,484,366	0.149	0.10	0.840	-	0.2	-	-0.020	2.1E-05
rs6733100	*LINC01320, LOC100288911*	intergenic	-	2p22.3	36,520,350	0.240	0.01	0.631	-	0.4	-	-0.017	2.2E-05
rs6056373	*PLCB1, PLCB4*	intergenic	-	20p12.3	9,040,569	0.101	0.79	0.138	-	0.7	-	0.023	2.8E-05
rs11770740	*COL26A1*	intronic	-	7q22.1	101,102,046	0.125	0.43	0.156	-	0.8	-	-0.021	2.9E-05

Abbreviations: Freq. GWAS, allele frequency in the 917 Amish subjects included in the GWAS; Freq. $1000g_{(EUR)}$, allele frequency in Total European Ancestry population from 1000 Genomes; Freq. $ESP_{(EA)}$, allele frequency in European American population from the National Heart, Lung, and Blood Institute (NHLBI) Exome Sequencing Project (ESP); Enrich. $1000g_{(EUR)}$, enrichment of allele frequency in the 917 Amish subjects included in the GWAS compared to allele frequency in Total European Ancestry population from 1000 Genomes (Freq. GWAS/Freq. $1000g_{(EUR)}$); Enrich. $ESP_{(EA)}$, enrichment of allele frequency in the 917 Amish subjects included in the GWAS compared to allele frequency in European American population from the NHLBI ESP (Freq. GWAS/Freq. $ESP_{(EA)}$).
