# Supplementary material for: From Genotype to Phenotype: Nonsense Variants in SLC13A1 Are Associated with Decreased Serum Sulfate and Increased Serum Aminotransferases
Source: G3 (Bethesda). 2016 Jul 13;6(9):2909–18. doi: 10.1534/g3.116.032979 (PMC5015947; doi:10.1534/g3.116.032979)
Supplement: Supplemental Material [file supp_g3.116.032979_TableS7.pdf]

Table S7. Associations between sulfate-lowering SNVs, and bone mineral density (BMD) measurements.

Trait	Sulfate-lowering SNV(s)	n	Het.	Hom.	β_{SNV} ± SE	β_{SNV}/ $Mean_{WT}$	P_{SNV}
**Mid-arm BMD (g/cm^2)**	*SLC13A1* R12X or W48X	680	20	0	-0.02 ± 0.01	-0.04	0.08
	SLC26A1 L348P	646	70	6	-0.01 ± 0.01	-0.02	**0.03**
**Mid-arm Z-Score**	*SLC13A1* R12X or W48X	680	20	0	-0.45 ± 0.24	-0.48	0.07
	SLC26A1 L348P	646	70	6	-0.22 ± 0.12	-0.23	0.06
**1/3-arm BMD (g/cm^2)**	*SLC13A1* R12X or W48X	680	20	0	-0.03 ± 0.02	-0.04	0.08
	SLC26A1 L348P	646	70	6	-0.02 ± 0.01	-0.02	**0.03**
**1/3-arm Z-Score**	*SLC13A1* R12X or W48X	680	20	0	-0.50 ± 0.25	-0.55	**0.04**
	SLC26A1 L348P	646	70	6	-0.22 ± 0.12	-0.24	0.06
**Total-arm BMD (g/cm^2)**	*SLC13A1* R12X or W48X	680	20	0	-0.02 ± 0.01	-0.04	0.06
	SLC26A1 L348P	646	70	6	-0.02 ± 0.01	-0.02	**0.01**
**Total-arm Z-Score**	*SLC13A1* R12X or W48X	680	20	0	-0.47 ± 0.24	-0.51	0.05
	SLC26A1 L348P	646	70	6	-0.24 ± 0.11	-0.25	**0.04**
**Ultra-distal-arm BMD (g/cm^2)**	*SLC13A1* R12X or W48X	680	20	0	-0.02 ± 0.01	-0.03	0.26
	SLC26A1 L348P	646	70	6	-0.01 ± 0.01	-0.03	**0.03**
**Ultra-distal-arm Z-Score**	*SLC13A1* R12X or W48X	680	20	0	-0.29 ± 0.24	-0.40	0.23
	SLC26A1 L348P	646	70	6	-0.24 ± 0.12	-0.32	**0.04**
**Femoral neck BMD (g/cm^2)**	*SLC13A1* R12X or W48X	681	20	0	-0.02 ± 0.03	-0.02	0.46
	SLC26A1 L348P	647	70	6	-0.02 ± 0.01	-0.02	0.26
**Femoral neck Z-Score**	*SLC13A1* R12X or W48X	681	20	0	-0.20 ± 0.24	-0.37	0.42
	SLC26A1 L348P	647	70	6	-0.12 ± 0.11	-0.23	0.30
**Intertrochanter BMD (g/cm^2)**	*SLC13A1* R12X or W48X	680	20	0	-0.04 ± 0.04	-0.03	0.35
	SLC26A1 L348P	646	70	6	-0.05 ± 0.02	-0.04	**$5.1x10^{-3}$**
**Intertrochanter Z-Score**	*SLC13A1* R12X or W48X	680	20	0	-0.24 ± 0.22	-0.50	0.29
	SLC26A1 L348P	646	70	6	-0.30 ± 0.11	-0.62	**$5.2x10^{-3}$**
**Total-hip BMD (g/cm^2)**	*SLC13A1* R12X or W48X	681	20	0	-0.03 ± 0.03	-0.03	0.28
	SLC26A1 L348P	647	70	6	-0.04 ± 0.01	-0.04	**0.01**
**Total-hip Z-Score**	*SLC13A1* R12X or W48X	681	20	0	-0.29 ± 0.23	-0.49	0.21
	SLC26A1 L348P	647	70	6	-0.27 ± 0.11	-0.45	**0.02**
**Trochanter BMD (g/cm^2)**	*SLC13A1* R12X or W48X	680	20	0	-0.03 ± 0.03	-0.04	0.23
	SLC26A1 L348P	646	70	6	-0.03 ± 0.01	-0.03	**0.04**
**Trochanter Z-Score**	*SLC13A1* R12X or W48X	680	20	0	-0.32 ± 0.23	-0.53	0.17
	SLC26A1 L348P	646	70	6	-0.23 ± 0.11	-0.38	**0.04**
**Total-spine BMD (g/cm^2)**	*SLC13A1* R12X or W48X	679	20	0	-0.03 ± 0.03	-0.03	0.39
	SLC26A1 L348P	645	70	6	-0.03 ± 0.02	-0.03	0.10
**Total-spine Z-Score**	*SLC13A1* R12X or W48X	679	20	0	-0.25 ± 0.29	-0.92	0.39
	SLC26A1 L348P	645	70	6	-0.19 ± 0.14	-0.67	0.18
**Whole-body BMD (g/cm^2)**	*SLC13A1* R12X or W48X	679	20	0	-0.03 ± 0.02	-0.03	0.19
	SLC26A1 L348P	645	69	6	-0.03 ± 0.01	-0.03	**$6.8x10^{-3}$**

Adjusted for age and gender. Abbreviations: Het., heterozygotes; Hom., homozygotes; WT, wild type; SE, standard error.
