# Supplementary material for: From Genotype to Phenotype: Nonsense Variants in SLC13A1 Are Associated with Decreased Serum Sulfate and Increased Serum Aminotransferases
Source: G3 (Bethesda). 2016 Jul 13;6(9):2909–18. doi: 10.1534/g3.116.032979 (PMC5015947; doi:10.1534/g3.116.032979)
Supplement: Supplemental Material [file supp_g3.116.032979_TableS8.pdf]

Table S8. Associations between serum sulfate, and serum alanine aminotransferase (ALT) and aspartate aminotransferase (AST) levels and bone mineral density (BMD) measurements.

Trait	SNV covariate(s)	n	β_{SNV} ± SE	P_{SNV}
**ALT (U/L)**	SO_4	663	-3.71 ± 4.31	0.39
**Mid-shaft radius BMD (g/cm^2)**	SO_4	660	0.01 ± 0.03	0.70
**Mid-shaft radius Z-Score**	SO_4	660	-0.12 ± 0.58	0.84
**Proximal (1/3rd) radius BMD (g/cm^2)**	SO_4	660	0.04 ± 0.04	0.23
**Proximal (1/3rd) radius Z-Score**	SO_4	660	0.63 ± 0.60	0.29
**Total forearm BMD (g/cm^2)**	SO_4	660	0.02 ± 0.03	0.55
**Total forearm Z-Score**	SO_4	660	0.10 ± 0.57	0.86
**Ultra-distal radius BMD (g/cm^2)**	SO_4	660	0.01 ± 0.03	0.80
**Ultra-distal radius Z-Score**	SO_4	660	-0.02 ± 0.57	0.98
**AST (U/L)**	SO_4	663	4.29 ± 3.01	0.15
**Calcium (mg/dL)**	SO_4	663	0.01 ± 0.18	0.97
**Corrected calcium (mg/dL)**	SO_4	663	0.08 ± 0.15	0.62
**Femoral neck BMD (g/cm^2)**	SO_4	661	0.01 ± 0.07	0.84
**Femoral neck Z-Score**	SO_4	661	-0.04 ± 0.57	0.95
**Intertrochanter BMD (g/cm^2)**	**SO_4**	**661**	**0.18 ± 0.09**	**0.04**
**Intertrochanter Z-Score**	SO_4	661	0.99 ± 0.52	0.06
**Total hip BMD (g/cm^2)**	SO_4	661	0.13 ± 0.07	0.08
**Total hip Z-Score**	SO_4	661	0.88 ± 0.055	0.11
**Trochanter BMD (g/cm^2)**	**SO_4**	**661**	**0.13 ± 0.06**	**0.03**
**Trochanter Z-Score**	SO_4	661	1.09 ± 0.54	0.04
**Lumbar spine BMD (g/cm^2)**	SO_4	659	0.07 ± 0.08	0.39
**Lumbar Spine Z-Score**	SO_4	659	0.50 ± 0.68	0.47
**Whole-body BMD (g/cm^2)**	SO_4	659	0.04 ± 0.06	0.46

Adjusted for age and gender. Abbreviations: SO_4, sulfate; SE, standard error.
