# Supplementary figures and images for: From Genotype to Phenotype: Nonsense Variants in SLC13A1 Are Associated with Decreased Serum Sulfate and Increased Serum Aminotransferases
Source: G3 (Bethesda). 2016 Jul 13;6(9):2909–18. doi: 10.1534/g3.116.032979 (PMC5015947; doi:10.1534/g3.116.032979)

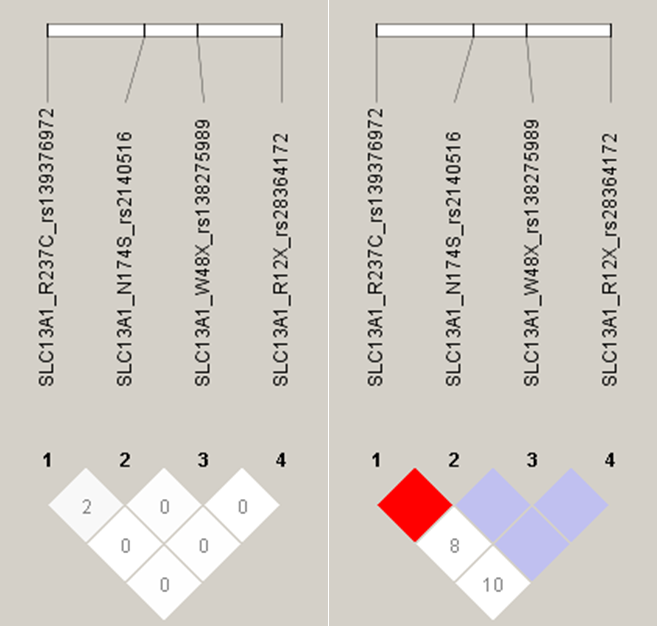

Supplement: Supplemental Material [file supp_g3.116.032979_FigureS1.tif]

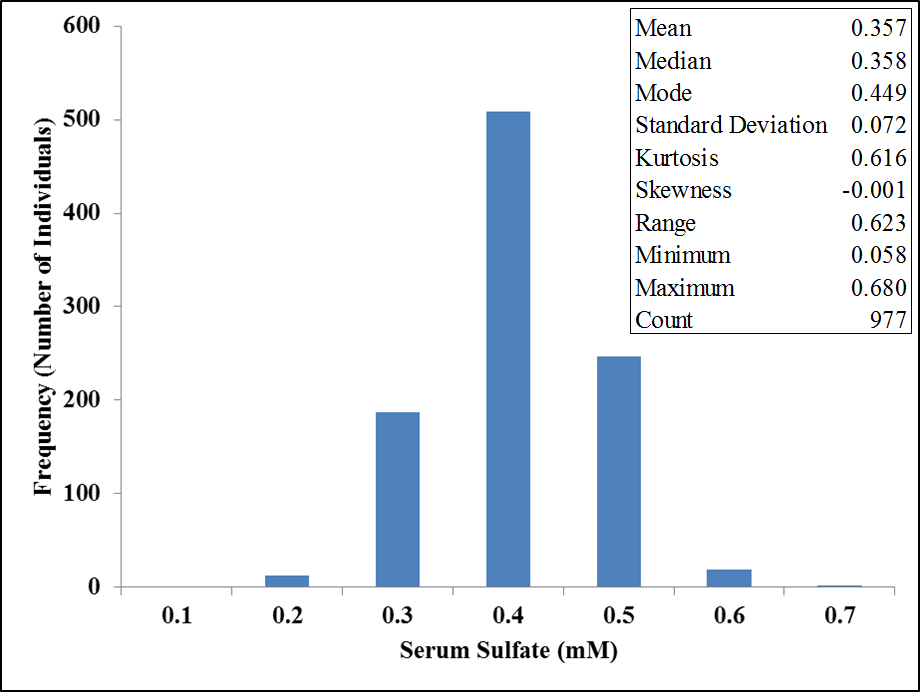

Supplement: Supplemental Material [file supp_g3.116.032979_FigureS2.tif]

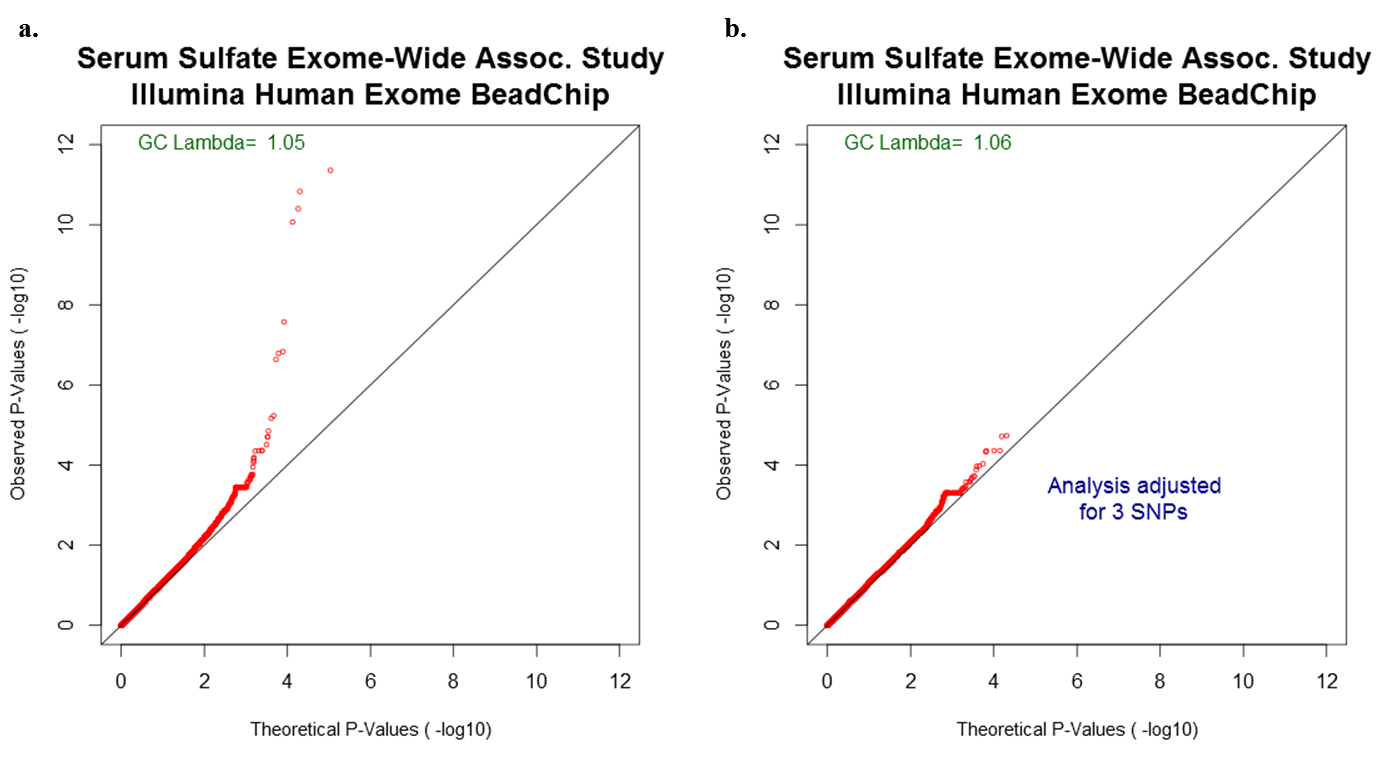

Supplement: Supplemental Material [file supp_g3.116.032979_FigureS5.tif]

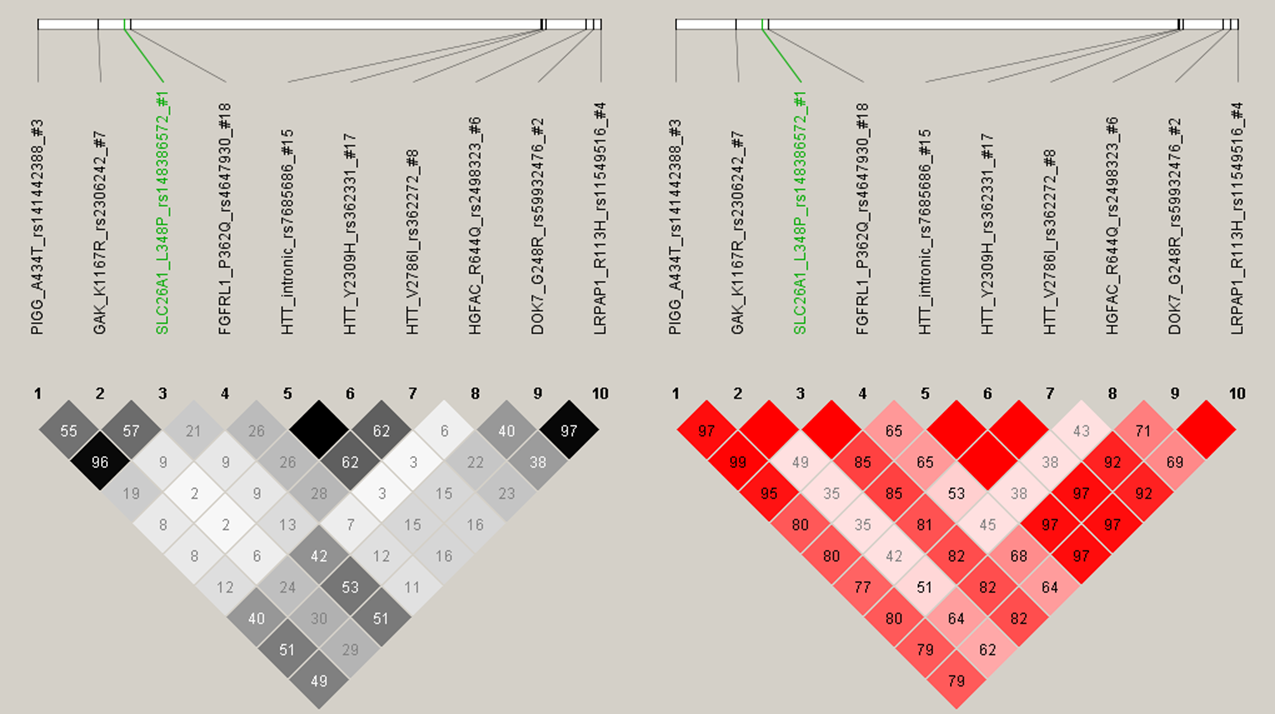

Supplement: Supplemental Material [file supp_g3.116.032979_FigureS6.tif]

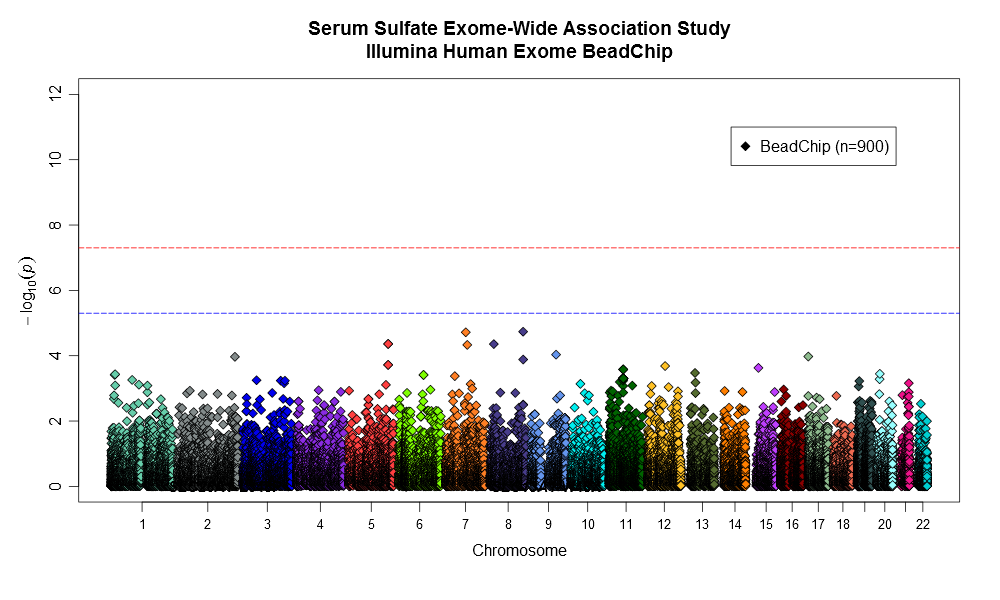

Supplement: Supplemental Material [file supp_g3.116.032979_FigureS7.tif]

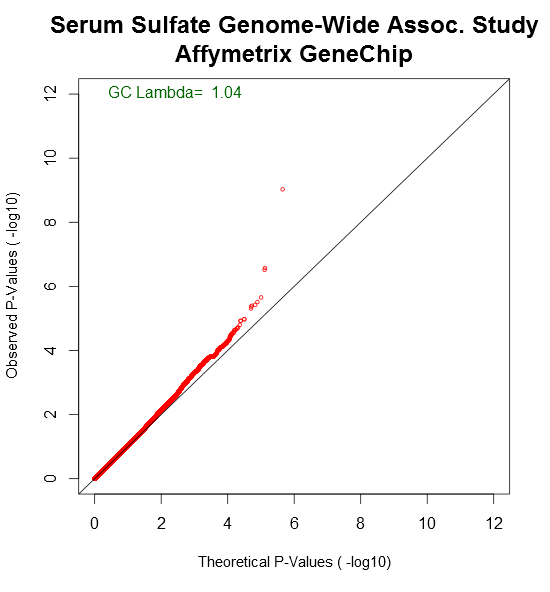

Supplement: Supplemental Material [file supp_g3.116.032979_FigureS8.tif]

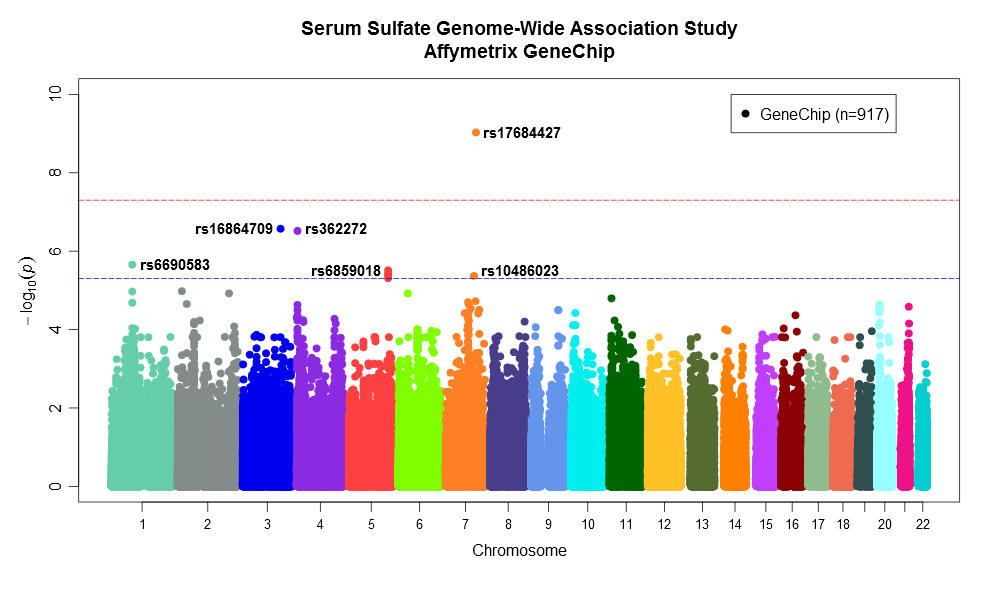

Supplement: Supplemental Material [file supp_g3.116.032979_FigureS9.tif]

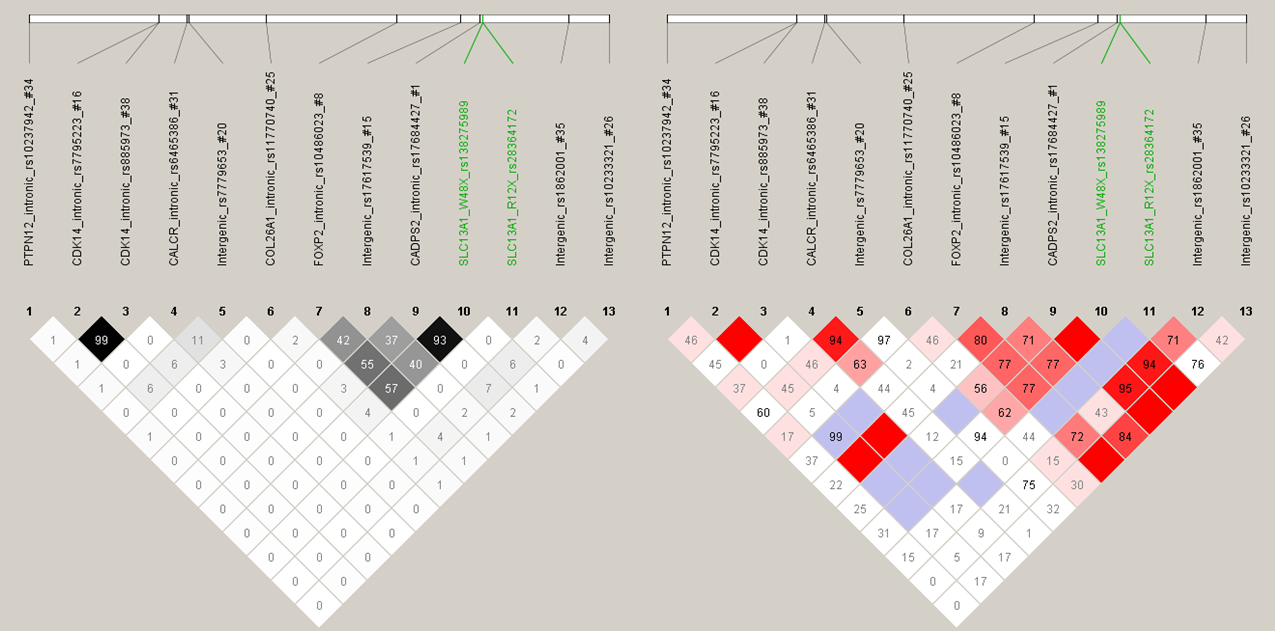

Supplement: Supplemental Material [file supp_g3.116.032979_FigureS10.tif]

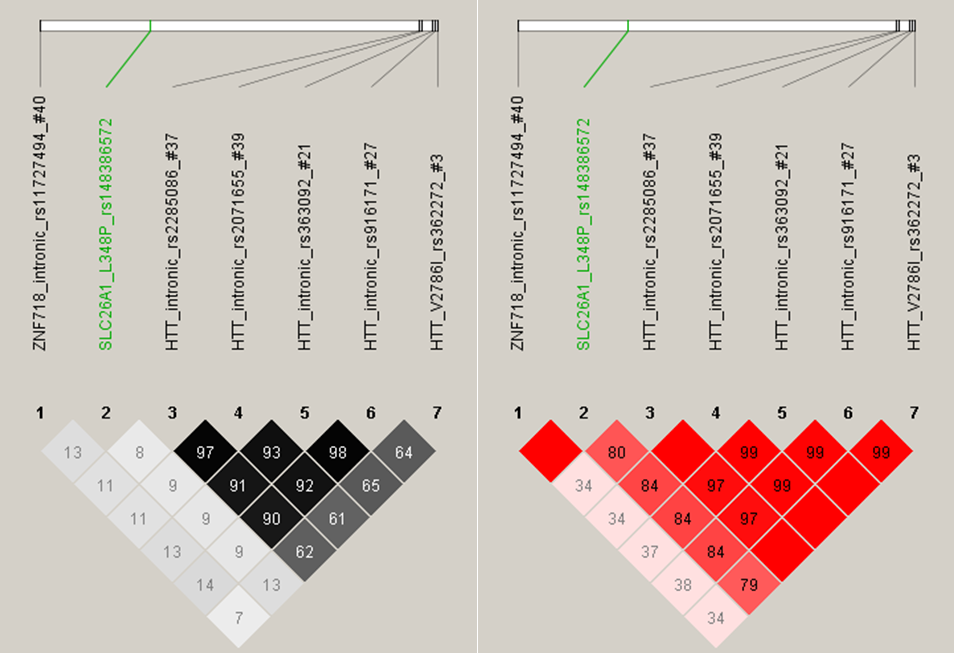

Supplement: Supplemental Material [file supp_g3.116.032979_FigureS11.tif]

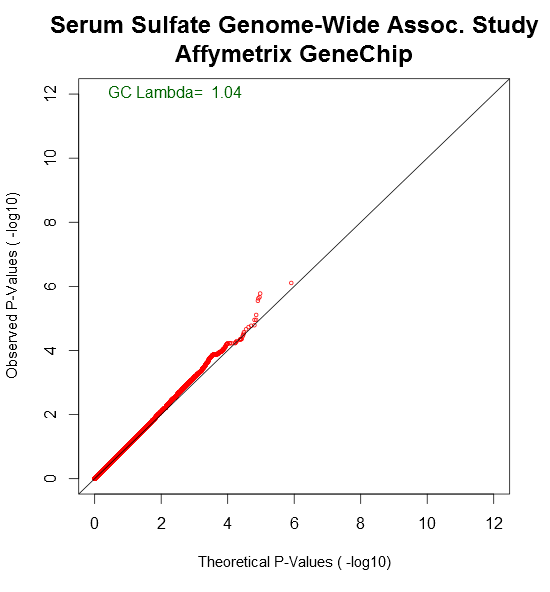

Supplement: Supplemental Material [file supp_g3.116.032979_FigureS12.tif]

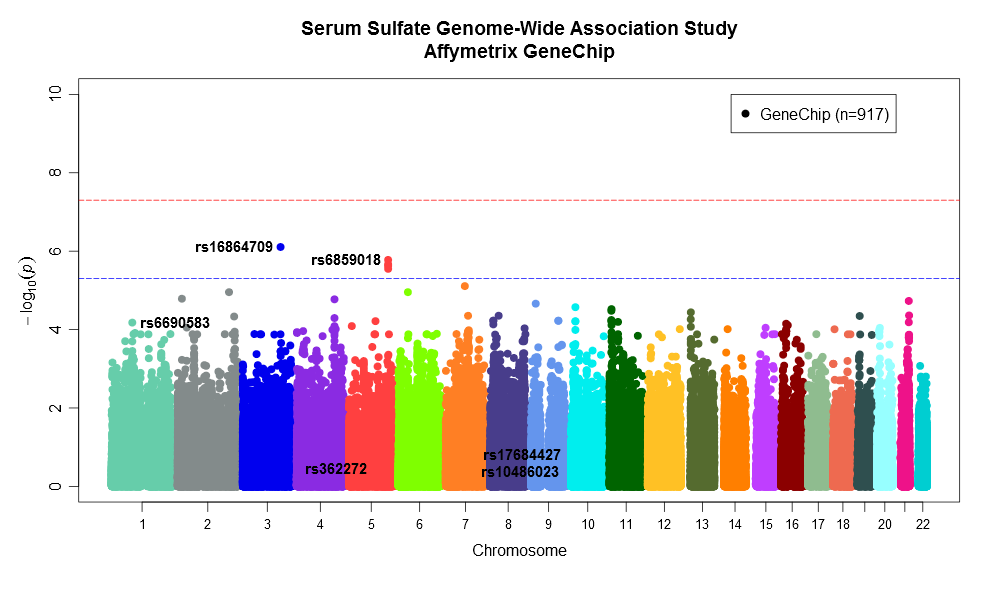

Supplement: Supplemental Material [file supp_g3.116.032979_FigureS13.tif]
